# Addictive-like behavioural traits in pet dogs with extreme motivation for toy play

**DOI:** 10.1038/s41598-025-18636-0

**Published:** 2025-10-09

**Authors:** Alja Mazzini, Katja Senn, Federico Monteleone, Stefanie Riemer

**Affiliations:** 1 Division of Animal Welfare, Vetsuisse Faculty, Veterinary Public Health Institute, University of Bern, Bern, 3012 Switzerland; 2 Division of Behavioural Ecology, Institute of Ecology and Evolution, University of Bern, Hinterkappelen, 3032 Switzerland; 3 Messerli Research Institute, Department of Interdisciplinary Life Sciences, Vetmeduni Vienna, Vienna, 1210 Austria

**Keywords:** Dogs, Behavioural addiction, Play behaviour, Toy play, Play motivation, Addiction criteria, Psychology, Motivation, Reward

## Abstract

Behavioural addictions, characterised by compulsive engagement in rewarding activities despite adverse consequences in the long term, are more heterogeneous and less well-understood than substance addictions, and there is a relative lack of translational research. This study investigates “excessive toy motivation” in domestic dogs as a potential parallel to behavioural addictions in humans. Employing a combination of a behavioural test and an owner questionnaire, we examined whether a subset of highly play-motived dogs meet key behavioural addiction criteria, including craving, salience, lack of self-control, and mood modification. Data from 105 highly play-motivated dogs revealed that 33 subjects exhibited behaviours consistent with addictive-like tendencies, including an excessive fixation on toys, reduced responsiveness to alternative stimuli, and persistent efforts to access toys. Owner-reported behaviours not only corroborated these findings but also demonstrated significant associations with behavioural test scores. Our results highlight parallels between excessive toy motivation in dogs and human behavioural addictions, with dogs as the only non-human species so far that appears to develop addictive-like behaviours spontaneously without artificial induction. This exploratory study provides foundational insights and proposes future research directions that have the potential to significantly deepen our understanding of the psychological mechanisms underlying behavioural addictions across species.

**Supplementary Information:**

The online version contains supplementary material available at 10.1038/s41598-025-18636-0.

## Introduction

What is play? Why do many large-brained mammals engage in play throughout their lives? And what makes playing potentially addictive? Despite numerous publications on play and its possible functions, play has remained somewhat of a mystery^1^, being associated with no immediate adaptive function, although it has been suggested to allow animals to practice species-typical behaviours such as hunting, mating, or fighting with a competitor in a non-serious context^1^. Notwithstanding, play behaviour is ubiquitous among (at least young) mammals and some birds^2,3^, and in large-brained species in particular – from humans to dogs – it persists throughout life^4,5^. Still, no unified definition of play exists to date^6^, although there is some agreement that, at a proximate level, play makes us feel good^7^. Even in non-human animals, observers will often agree that playful activities look like fun^7^, with neurotransmitter systems mediating the rewarding aspects of play (opioids, cannabinoids and dopamine)^8,9^ appearing to be highly conserved across mammalian taxa^10^.

Bateson (2014) proposed a set of characteristics that are generally accepted to define play: it is spontaneous, intrinsically rewarding and “fun”; it is separate from serious consequences; it often involves novel or exaggerated actions and role reversal; it is repetitive, but distinct from stereotypies; and it usually occurs only in healthy, stress-free animals, making it a marker of well-being^7^. However, regarding the latter point, it has been highlighted that play can also represent an attempt to cope with suboptimal conditions (e.g. in nonhuman animals, play may occur as displacement behaviour in stressful situations^5^ or may serve to reduce social tensions)^11^. Also, in humans, playing computer games or gambling represents a way of coping with stress. Moreover, in some instances, what started as a fun activity can become compulsive and develop into a behavioural addiction^12–14^.

A behavioural addiction can be defined as “repeated failure to resist an impulse, drive, or urge to perform an act that is rewarding to the person (at least in the short-term), despite longer-term harm to the individual or others” (ICD-11 (International Classification of Diseases 11th Revision))^15^. Unlike in compulsive disorders, where performance of the compulsive behaviour primarily serves to provide some relief from a negative affective state, i.e. via negative reinforcement, addictive behaviours originate because their performance generates positive affect, i.e. via positive reinforcement. However, as the addiction develops, the behaviour becomes compulsive and may even cease to be rewarding (reviewed by Freimuth et al.^16^. Behavioural addictions share underlying neurobiological processes^17^ and behavioural symptoms (such as craving, lack of self-control, tolerance, withdrawal and risk of relapse) with substance addictions^17,18^. Still, they are more heterogeneous and less well-understood^19^.

While a wide range of behaviours have the potential to become addictive in people (e.g. exercise, sex, shopping, work, etc.)^19–25^, to date, only the two disorders related to playing – gambling and internet gaming – are officially recognised as behavioural addictions in the two psychiatric manuals of psychological disorders (DSM-5 and ICD-11). The ICD-11^15^ included both gambling and internet gaming as behavioural addictions. In the 5th edition of DSM-5^26^, gambling, previously classified as an impulse control disorder, was included under “substance-related and addictive disorders”^19,26^, while internet gaming was listed separately as “internet gaming disorder”^26^.

What would make behaviours related to playing so addictive? Play involves neurotransmitter systems (opioids, cannabinoids, and dopamine) that are also engaged in the rewarding aspects of food and drug rewards^8,9^. Thus, video games can provide players a hedonic experience and a high degree of relaxation^27^. Pathological gaming is an example of how seemingly normal and enjoyable behaviours can develop to disrupt regular social and environmental functioning^28–30^.

Compared to substance addiction, there are only a few animal models of behavioural addictions. Moreover, these are restricted to controlled laboratory settings, and addictive-like behaviour has to be actively induced^31^. Rodent models have been used to investigate compulsive eating (e.g. reviewed in^31^, exercise addiction (wheel running^32,33^), gambling^34^, and responses to sexual reward^35^. Mice selectively bred for excessive wheel-running, sometimes referred to as an addiction-prone phenotype, develop physiological withdrawal symptoms similar to those found in drug addiction after abstinence^36^. As with excessive exercise in humans, wheel-running in rodents may become disruptive to everyday activities, leading to impaired nest-building and sheltering behaviour^37,38^. The animals may continue to wheel-run despite disrupted sleep^39^ or even in the face of injury^40^, thus fulfilling the behavioural addiction criterion “persistence of the behaviour despite adverse consequences”^41^. This suggests that behavioural addictions are not unique to the human species.

There is, however, only one species that appears to display addictive-like behaviour spontaneously, without intentional experimental induction: the domestic dog (although inadvertent promotion of addictive-like behaviour by the caretakers cannot be ruled out). A small subset of dogs – colloquially referred to as “ball junkies” – appear to demonstrate an addictive-like desire for object play^42^.

Like humans, domestic dogs frequently remain playful throughout their lives^1^, engaging in both social and object-related play, as well as combinations (e.g. tug-of-war^9^. Solitary object play appears to be related to predatory behaviour^9^; accordingly the development of social and object play may reflect different selective histories of dog breeds, which were selected for various purposes such as hunting, guarding, herding, and other functions^6–8^.

Toy play is a potent reinforcer, especially in working dog training^43–45^. For instance, detection dogs working in public settings are typically not rewarded with food due to concerns about undesirable food-seeking behaviours in the field. Still, they will work persistently for their toy rewards. It has been argued that artificial selection has exaggerated play behaviour in adult dogs, especially in working breeds or working lines, where high toy motivation is often actively selected as a predictor of performance^46^. For example, in Labrador retrievers, working lines demonstrate higher playfulness than show lines, indicating a genetic basis for play motivation and potential for artificial selection^47^.

Playing with toys allows dogs to express instinctive predatory sequences such as chasing, catching, possessing and “dissecting”, considered to be intrinsically rewarding to them based on their species and breed histories^48^. None of this is pathological, nor is gambling or computer gaming in people. However, such highly rewarding activities have the potential to become obsessive in humans^49,50^, and the same may be true for dogs.

While addictive-like behaviour towards toys in dogs has not been studied to date, the phenomenon has been described in the lay literature (where affected dogs may be referred to as ‘ball junkies’), and it has been (rarely) alluded to in the scientific literature. Lazarowski et al.^48^ describe how some dogs show behavioural and physiological signs of high arousal in relation to toys, lack of self-control, and behaviours such as whining, barking, spinning, and other behavioural signs of stress when access to a toy is prevented (e.g. because the dog is restrained), suggested as an expression of their inability to manage the frustration of anticipation^46^. All these signs could be interpreted as indicative of craving (and frustration when the urge cannot be fulfilled).

In humans, addictive behaviours are often associated with deficits in inhibitory control and heightened cue-reactivity and craving, which are likely key mechanisms in addiction, particularly when exposed to behaviour-specific cues^51–53^.

In animal models of addictions, not only is an increased motivation to work for the rewarding substance notable, but the animals also continue seeking the reward even when it is signalled to be unavailable^54^. Similarly, excessively toy-motivated dogs may continue to try to gain access to a toy even when the caretaker has put it away (anecdotal evidence^55^. Dogs that appear obsessed with toys cannot be easily distracted from their fixation on the preferred object – demonstrating the high salience of the toy. Such dogs may even lose interest in other stimuli or social interactions as long as they have access to the toy, or sometimes even when it has been removed from reach^55^ – i.e., everyday functioning may be affected. Moreover, some dogs may continue playing (e.g., running tirelessly after balls thrown for them) despite adverse consequences, such as over-exertion or even injury in the short term and damage to joints and ligaments in the longer term^56^.

Thus, we suggest that ‘excessive toy motivation’ in dogs may show parallels to behavioural addictions in humans. Domestic dogs share many complex behavioural traits with us^57,^and they are commonly used as model species to explore compulsive behaviours^58,59^; cognitive ageing^60–62^, ADHD^63–65^, neuroticism^66^ and autism^67–69^.

## Rationale

Here, we aim to provide the first scientific evaluation of ‘excessive toy motivation’ in dogs, develop methods to assess this phenomenon, and investigate whether ‘excessive toy motivation’ in pet dogs meets the defining criteria of behavioural addictions. Due to the heterogeneity of behavioural addictions, the number and description of diagnostic criteria are inconsistent in the scientific literature, even in humans^19^. We decided to explore whether the most common behavioural addiction criteria can be adapted to dogs: (1) craving, (2) salience, (3) mood modification through carrying out the behaviour, (4) lack of self-control, (5) tolerance, (6) withdrawal symptoms, (7) external consequences (the addictive behaviour causes conflict with other activities, other individuals, or within the individual), and (8) relapse after abstinence from the activity (cf^28,41,70,71^. Two additional criteria are used for diagnosing behavioural addictions in humans: having problems at home or work and lying to/deceiving people close to them^26,72^. Since these criteria cannot be applied to animals, we focused only on the eight abovementioned criteria.

We developed a behavioural test exposing pet dogs to various situations where behavioural addiction criteria in relation to toys can be expressed. Only the first four of the criteria mentioned above can be measured in a single behavioural assessment. The remaining criteria were included in an accompanying questionnaire, in which the dogs’ owners were asked about their dogs’ everyday behaviour.

While this study is exploratory, given the lack of prior research in this area, we used convergent methodologies in an attempt to assess internal and external validity. We predicted that dogs classified as having a high tendency for addictive-like behaviour based on our continuous Addictive-like Behaviour Test score, would:

- Show higher scores for the individual behavioural addiction criteria: Salience, Craving, Mood modification, and Lack of self-control in the behaviour test,
- Show higher durations of focusing on and trying to access an unavailable toy in the behaviour test,
- Receive higher scores on the owner questionnaire designed to measure dogs’ addictive-like behaviour in everyday life,

than dogs classified as having a low tendency for addictive-like behaviour.

## Methods

### Play motivation test

#### Ethical consideration

The study was assessed and approved by the Veterinary Office of the Canton of Bern, Switzerland (Licence number BE115/17). All procedures were performed in accordance with the “Guidelines for the Treatment of Animals in Behavioral Research and Teaching” of the Association for the Study of Animal Behavior. All dog owners provided written informed consent for their participation.

#### Subjects

One hundred twenty-six dog-owner teams were recruited via advertisements on social media. In the first call, any play-motivated dog was welcome to participate. In a second call, we specifically sought dogs showing ‘excessive’ motivation for toy play.

Twenty-one of the 126 tested dogs were excluded from the analysis as they were (1) outside the target age range (< 1 year or > 10 years old; *N* = 9), (2) did not complete the test due to fatigue (*N* = 1), (3) did not play at all or were too fearful for pulse measurements (*N* = 7), or (4) due to disturbances during the test (e.g. owners bringing young children along, *N* = 4). The final sample (*N* = 105) included 56 males (34 neutered or chemically castrated, 20 intact and 2 cryptorchids) and 49 females (34 neutered and 15 intact), ranging in age from 12 months to 10 years (mean age = 5.09 years, SD = 2.6). The dogs belonged to various breeds (for demographics, see Supplementary Table 1). Eighty-two owners (72 women and 10 men) participated in the study.

#### Experimental set-up

**Fig. 1:**
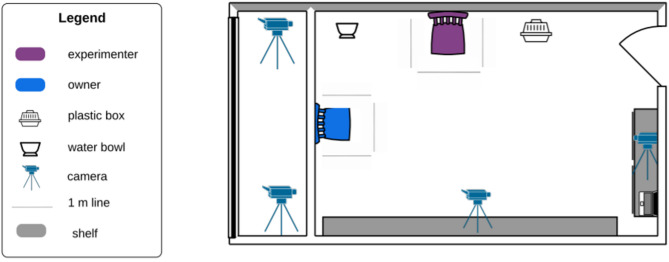
Experimental room with the experimenter and owner in the starting position.

Behaviour tests took place in an experimental room (Fig. 1), measuring 5.22 m x 3.36 m. A wooden partition wall divided the room into two parts so that the effective testing space was 3.60 m x 3.36 m. The room was furnished with two chairs and several shelves on the walls. One of the chairs was placed in front of the wooden partition wall (facing the entrance door), and the other was placed at a 90° angle against the wall to the left. In front of both chairs, a taped line marked a one-meter distance from the chairs. During the habituation phase, the opaque box in which a toy or food was enclosed during several subtests (hereafter, unsolvable task box) was placed next to the experimenter’s chair.

Four video cameras (IB8377-H; 4 MP, 30 fps, H.264, WDR Pro, IR, PoE, IP66, 2.8–12 mm) were placed in the room, and recordings were made using the recorder system (ND9441P NVR, 16-CH, 4HDD, H.265, HDMI/VGA, 16x PoE).

#### Methods

The test battery consisted of 14 subtests assessing various aspects of toy motivation in dogs. Play behaviour per se cannot be used to infer addictive-like behaviour, which is characterised primarily by reactions when the reward is unavailable; therefore, only subtests relevant to exploring behavioural addiction criteria are described in detail hereafter. The complete description of the play motivation test is available under: https://figshare.com/s/dfd6d12d922f7543b96c.

##### Procedure

###### Room habituation

After the owner and the dog had entered the test room, the dog was unleashed, and a 3-minute habituation phase commenced (Fig. 1). Meanwhile, the owner and the experimenter were seated on their allocated chairs, and the experimenter explained the test procedure. The owner signed the consent form. The owners were instructed to interact with the dog only when asked to perform one of the subtests and not to use food during testing unless absolutely necessary (such as exchanging food for a toy if the dog was unwilling to relinquish it).

###### Choosing the toy

After the habituation phase, the experimenter retrieved a box containing various commercial dog toys of different sizes and textures, with and without squeakers, etc., from the adjacent storage room. Only toys that might be associated with food enrichment were excluded. The owner was asked to select three toys (one ball, one tug toy and one plush toy) which they thought the dog would like the most. If the owner had brought the dog’s favourite toy from home, this toy was used in the subsequent preference test along with two other toys. 

After removing the toy box from the room, the experimenter returned to the test room. The owner recalled the dog and sat down on their chair, holding the dog behind the Line marking the 1 m distance from the chair. Opposite the dog at the front of the room, the experimenter placed the three toys on the floor in a row, 40 cm apart. After the experimenter had returned to her chair, the owner released the dog, who could now explore and play with the toys for 30 s. The two people present did not interact with the dog during this time. The toy the dog spent the most time interacting with was used for subsequent testing. Forty-five dogs selected a ball, nine selected a tug toy, 39 selected a plush toy, and 12 selected a hybrid toy (plush ball: N = 3; tug with a ball: N = 6; plush tug: N = 3). On rare occasions, the dog did not show interest in any of the toys. In this case, the owner was asked to choose the type of toy the dog was usually most interested in at home. The chosen toy was used throughout the experiment, and the remaining two toys were placed on the shelf out of reach and sight of the dog. If the preferred toy was not a tug toy, the tug toy was used in subtests where the owner or experimenter played tug-of-war with the dog.

##### Description of the subtests and their relevance for addiction criteria coding

A description of the subtests and, when applicable, their relevance for addiction criteria coding is given in Table 1.

**Table 1:** Description of subtests of the play motivation test and their relevance for addiction criteria, where applicable.

Subtests description	Relevance for addiction criteria coding
1. **Choosing the toy** The owner is asked to hold the dog behind the 1 m line. Three types of toys (tug, ball, and plush) are placed on the floor (40 cm apart). After the dog is released, she/he has 30 s to interact with the toys.	The dog’s eagerness to approach the toys prior to being released and mood modification when given access to the toys.
2. **Social play with toys** The owner is asked to play with the dog and the selected toy as they usually would for 1 min.	N/A
3. **Social play without toys** The owner engages the dog in social play (without toys) for 1 min while the toy used in the previous sequence is on a shelf (behind the experimenter), inaccessible to the dog.	The dogs’ ability to engage and focus on social play with the owner while the toy is out of reach.
4. **Tug-of-war** The owner is asked to engage the dog in a tug game for 1 min.	N/A
5. **Solitary play after the tug-of-war** The owner is asked to let the dog win the tug-of-war game and keep the tug toy for another 30 s without further interaction. Next, the experimenter removes the tug toy (if the preferred toy is not a tug toy) and places the preferred toy on the floor. The dog has free access to the toy for 1.5 min.	N/A
6. **Dog alone** The toy is available to the dog; the owner and experimenter leave the room for 30 s.	Engaging with a toy despite adversity (being left alone in an unfamiliar room is stressful for most dogs) ^73–75^.
7. **Solitary play after being alone** The owner and experimenter returned after 30 s, and the dog continued to have access to the toy for 1.5 min.	N/A
8. **Thrown toy** First, the experimenter holds the preferred toy in her hand for a few seconds, showing it to the dog. Then, she throws the toy without any social interaction with the dog. The dog is off-lead and can chase the toy. After each throw, the experimenter or, if necessary, the owner asks the dog to release the toy. After the experimenter has thrown the toy three times, the owner repeats the procedure. In the next step, the experimenter throws the toy three times while motivating the dog to play verbally and with her body language. Subsequently, the owner does the same.	Self-control before the toy is thrown; the dog’s willingness to relinquish the toy after each throw.
9. **Food puzzle** In full view of the dog, the experimenter places 10 pieces of dry food in the food puzzle (meanwhile, the toy is on the shelf behind the experimenter). The dog is released and has 3 min to learn how to obtain the food. The owner can help and encourage the dog if needed.	The dogs’ capability to focus on learning how to obtain food from the puzzle while the toy is out of reach (on the shelf).
10. **Toy inaccessible (in a box)** The toy is placed into the unsolvable task box, thus rendering it inaccessible to the dog. The food puzzle, filled with 5 pieces of dry food, is placed at a distance of 40 cm from the box, freely available to the dog. The dog is released and has 3 min of free interaction with the box or the food puzzle. The owner and the experimenter do not interact with the dog.	Willingness to engage in alternative behaviour (getting food out of the food puzzle) while the toy is inaccessible; effort to obtain the toy.
11. **Food puzzle inaccessible (in a box)** The food puzzle with 5 pieces of dry food is placed into the unsolvable task box, and the toy is placed on the floor at a distance of 40 cm, freely available to the dog. During the 3 min of the subtest, the owner and the experimenter do not interact with the dog.	N/A
12. **Toy vs. food puzzle** The toy and the food puzzle, filled with 5 pieces of dry food, are placed on the floor, 40 cm apart, and are available for the dog for 3 min. The owner and the experimenter do not interact with the dog.	N/A
13. **Toy on a shelf** The toy is placed out of the dog’s reach on a shelf (opposite the experimenter) for 3 min. The owner and the experimenter do not interact with the dog.	Dog’s ability to relax or engage in other behaviours (exploring, lying down, etc.) when the toy is inaccessible vs. focus on the toy.
14. **Cool-down period** After the active part of the test, all toys and food are removed from the room. The owner and the experimenter are sitting on their designated chairs. For a duration of 15 min, the only interaction with the dog is the experimenter measuring the dog’s pulse every 5 min.	Dog’s ability to relax or engage in other behaviours (exploring, laying down, etc.) when the toy has been removed from the room.

#### Behavioural coding

Videos were coded using Solomon Coder (Solomon Coder beta 19.08.02, Copyright 2006–2019 by Andràs Péter).

For most subtests, the starting point for coding was when the experimenter and the owner were sitting on their chairs, and the dog was behind the Line, which marked a 1 m distance from the owner’s chair (Fig. 1).

Qualitative and quantitative coding was performed by coders who were not involved in the experiments.

Three different coding approaches were employed:

- Scoring of individual variables that may be indicative of addictive behaviour each minute, which were later summed up as Addictive-like Behaviour Test score (Table 2); coder: KS.
- Coding of presence/absence of the four addiction criteria in each minute of each subtest; coder: KS.
- Quantitative coding.

- Scoring and point sampling of behaviours during the subtests “Social play” and “Dog alone” (Table 4); coder: FL.
- Coding of absolute durations of behaviours in subtests where the toy was rendered inaccessible (unsolvable task box and toy on a shelf, see Table 4); coders: DZ and AH.

A second coder (AM) performed reliability coding of addictive-like behaviours and behavioural addiction criteria and point sampling for 15 dogs. Reliability between the two coders who coded the durations was also analysed for 15 dogs. Reliability was good or excellent for all included variables (ICC, absolute agreement, single measures, two-way mixed-effects model, computed in IBM SPSS Statistics Version 23 (IBM Corporation and its Licensors 1989, 2015) (see Supplementary Table 3 for full results).

#### Sub-criteria to generate an Addictive-like behaviour test score (AB-T score)

To quantify dogs’ propensity for addictive-like behaviour as objectively as possible, we introduced the Addictive-like Behaviour Test score (AB-T score). Applicable sub-criteria were rated for each minute of the test, and for analysis, each sub-criterion was assigned a score between 0 and 2 points, as detailed in Table 2. The points from all the subtests, including the cool-down period, were added to yield the AB-T score. The maximum possible value of the AB-T score was 120 points. A cut-off point for addictive-like behaviour was defined by a data range split divided into two halves. Dogs scoring equal to or above the mid-point (44.2 points) are referred to as dogs showing a high tendency for addictive-like behaviour or high-AB dogs.

Dogs scoring less than 44.2 points are referred to as low-AB dogs (dogs with a low tendency for addictive-like behaviour). The sub-criteria included in the Addictive-like Behaviour Test score (as detailed in Table 2) were selected as they were assumed to be relatively independent of the level of obedience and training. Dogs might have been trained to drop a toy on a cue and to exert impulse control and refrain from jumping towards the toy in the experimenter’s hand; therefore, these variables were not included in the AB-T score. However, behaviours such as staring at the toy or pacing are believed to be less subject to training and were included.

**Table 2:** Variables included in the AB-T score and representation of the scoring system.

Subtest	Variable name	Definition and Score
**3. Social play** (Owner playing with the dog without toys while the toy is on a shelf)	Focus on toy	⋅ Not focused on the toy = 0 points ⋅ Focused on the toy < 50% = 1 point ⋅ Focused on the toy > 50% = 2 points
	Arousal towards toy	⋅ Low = 0 points ⋅ Medium = 1 point ⋅ High = 2 points
	Vocalising towards toy	⋅ Yes = 2 points ⋅ No = 0 points
	Jumping towards toy	⋅ Yes = 2 points ⋅ No = 0 points
**6. Dog alone** (The toy is available to the dog; owner and experimenter leave the room for 30 s)	Playing with the toy (despite adverse consequences)	⋅ Not interacting with the toy = 0 points ⋅ Playing part of the time (< 50%) = 1 point ⋅ Playing most of the time (> 50%) = 2 points
**9. Food puzzle** (Dry food in the food puzzle, the dog has 3 min to learn to extract the food)	Eat *(over all 3 min)*	⋅ All food consumed = 0 points ⋅ Part of the food consumed = 0.5 points ⋅ No food consumed = 1 point
	Focus on the toy on the shelf *(points awarded for each minute)*	⋅ Not focused on the toy = 0 points ⋅ Focused on the toy < 50% of time = 1 point ⋅ Focused on the toy ≥ 50% of time = 2 points
	Pacing *(for each minute)*	⋅ Repetitive movement at a higher pace without an obvious goal, not exploring or sniffing ⋅ Yes = 2 points ⋅ No = 0 points
	Arousal towards toy *(for each minute)*	⋅ Low = 0 points ⋅ Medium = 1 point ⋅ High = 2 points
	Jumping towards the toy *(for each minute)*	⋅ Yes = 2 points ⋅ No = 0 points
	Vocalising *(for each minute)*	⋅ Yes = 2 points ⋅ No = 0 points
**10. Toy inaccessible in a box** (The dog’s toy is placed in a closed box, rendering it inaccessible, for 3 min. The food puzzle is freely available).	Focus on the box *(points awarded for each minute)*	⋅ Not focused on the box = 0 points ⋅ Focused on the box < 50% = 1 point ⋅ Focused on the box > 50% = 2 points
	Effort to get into the box (for each minute)*	⋅ Low = 0 points ⋅ Medium = 1 point ⋅ High = 2 points
	Vocalising *(for each minute)*	⋅ Yes = 2 points ⋅ No = 0 points
	Arousal *(for each minute)*	⋅ Low = 0 points ⋅ Medium = 1 point ⋅ High = 2 points
	Pacing *(for each minute)*	⋅ Repetitive movement at a higher pace without an obvious goal, not exploring or sniffing ⋅ Yes = 2 points ⋅ No = 0 points
	Food consumption *(over all 3 min)*	⋅ All food consumed = 0 points ⋅ Part of the food consumed = 0.5 points ⋅ No food consumed = 1 point
**13. Toy on a shelf** (The toy is placed on a shelf out of the dog’s reach for three minutes)	Vocalising *(points awarded for each minute)*	⋅ Yes = 2 points ⋅ No = 0 points
	Focus on the shelf *(for each minute)*	⋅ Not focused on the toy (shelf) = 0 points ⋅ Focused on the toy (shelf) < 50% = 1 point ⋅ Focused on the toy (shelf) ≥ 50% = 2 points
	Arousal *(for each minute)*	⋅ Low = 0 points ⋅ Medium = 1 point ⋅ High = 2 points
	Pacing *(for each minute)*	⋅ Repetitive movement, without a goal at a higher pace, not exploring or sniffing ⋅ Yes = 2 points ⋅ No = 0 points
	Jumping towards the toy *(for each minute)*	⋅ Yes = 2 points ⋅ No = 0 points
	Going directly under the shelf and looking up intensely *(for each minute)*	⋅ Yes = 2 points ⋅ No = 0 points
**14. Cool-down period** (All toys and food are removed from the room; owner and experimenter do not interact with the dog for 15 min)	Focus on the door through which toys were removed *(points awarded for each minute)*	⋅ Yes = 0.1 points ⋅ No = 0 points
	Focus on either shelf where a toy was kept during previous subtests *(for each minute)*	⋅ Yes = 0.1 points ⋅ No = 0 points
	Arousal *(for each minute)*	⋅ Low = 0 points ⋅ Medium = 0.1 point ⋅ High = 0.4 points
	Pacing *(for each minute)*	⋅ Yes = 0.4 points ⋅ No = 0 points
	Vocalising *(for each minute)*	⋅ Yes = 0.4 points ⋅ No = 0 points

* High effort: the dog attempted to enter the box by either pawing at it or biting it, pushing it at least 50 cm in a single attempt, or scratching or digging at the box with both paws.

Low effort: the dog circled the box for less than 10 s and kept its nose or paw more than 5 cm away from the box.

#### Presence/absence of behavioural addiction criteria

Separately from the AB-T variables, in each subtest, the addiction criteria Salience, Craving, Mood modification, and Lack of self-control were rated each minute as present or absent based on the occurrence of pre-defined behaviours. For instance, Salience was inferred from searching for a toy although there was an »attractive alternative« (food, owner inviting the dog to play). Craving was based on the dog focusing mainly on the toy (> 50%) and medium to high arousal directed at the inaccessible toy (inferred from behaviours such as panting, restlessness, and high muscle tension). We further coded behaviours that are usually characteristic of high arousal in some dogs, e.g., pacing, jumping towards the toy, and vocalising (see Table 3). If at least one of the pre-defined behaviours was expressed, the respective addiction criterion was coded as present. Tolerance, Withdrawal symptoms and Risk of relapse after abstinence could not be tested in the setting of the play motivation test since they develop over time.

For each subtest, a summary score for each of the four addiction criteria was computed by summing up the points for each minute of the subtest.

**Table 3:** Behavioural addiction (BA) criteria coded as present or absent (1/0) for each subtest. A given BA criterion was coded as present in a given subtest if at least one of the indicators was observed (except for variables used only in combination).

Subtest	Behavioural addiction criterion	Indicators to code BA criterion as present
**1. Choosing the toy** (Dog is presented with three types of toys)	**Lack of Self-Control** (Dog is recalled behind the line)	1. Dog remains focused on toys despite owner’s recall 2. Dog tries to access toys in the experimenter’s hands despite the owner’s recall
	**Craving** (Dog is behind the line, held by the owner)	1. Pulling towards toys while restrained by the lead, collar or harness 2. Jumping towards toy 3. Vocalising 4. Medium to high arousal directed at the toy
	**Mood modification** (Dog has been released and has access to toys)	1. Fast movements 2. Pounce on toy 3. Medium to high arousal directed at the toy
**3. Social play** (Owner trying to engage the dog in social play while the toy is on the shelf)	**Salience**	1. Focus on the toy for more than 50% of the time, although there is an “attractive alternative”
	**Craving**	1. Focus on the toy > 50% of the time 2. Jumping towards toy 3. Vocalising towards the toy 4. Medium to high arousal (only in combination)
**6. Dog alone** (The toy is freely available; owner and experimenter leave the room for 30 s)	**Salience**	1. Dog is interacting with the toy (despite the owner’s absence)
**8. Throw toy** (Experimenter and owner are throwing the toy with or without social interaction with the dog)	**Lack of Self-control** (before the toy is thrown)	1. Jumping in the direction of the hand/toy before the toy is thrown 2. Owner needs to hold the dog back while the experimenter is holding the toy
	**Mood modification** (after the toy is thrown)	1. Fast movements in the direction of the toy 2. Pounce on toy 3. Medium to high arousal directed at the toy
**9. Food puzzle** (Dry kibble in the food puzzle; toy is inaccessible on a shelf)	**Salience**	1. Looking for the toy although there is an “attractive alternative” 2. Not eating
	**Craving**	1. Focus on the toy > 50% of the time 2. Jumping towards toy 3. Medium to high arousal directed at the inaccessible toy 4. Pacing 5. Vocalising (only in combination)
**10. Toy inaccessible (in a box)** (The dog’s toy is placed in a closed box, rendering it inaccessible. The food puzzle is freely accessible)	**Lack of Self-control** (Owner is holding the dog behind the line before the test starts)	1. Pulling towards the box 2. Medium to high arousal directed at the box 3. Vocalising
	**Salience**	1. Focus on the box or a shelf (where the toy was stored previously) > 50% of the time 2. Not eating
	**Craving**	1. Effort to get into the box 2. Medium to high arousal directed at the box 3. Vocalising (only in combination) 4. Pacing (only in combination)
**13. Toy on a shelf** (The toy is placed on a shelf out of the dog’s reach)	**Salience**	1. Focus on the toy > 50% of the time
	**Craving**	1. Go directly under the shelf and look up intensely 2. Jumping/climbing towards the toy 3. Vocalising towards the toy 4. Medium to high arousal (only in combination) 5. Pacing (only in combination)
**14. Cool-down period** (Toys and food are removed from the room)	**Craving**	1. Medium to high arousal 2. Vocalising at the door or shelf 3. Focus on the door or shelf (only in combination) 4. Pacing (only in combination)

#### Quantitative coding

Selected behaviours during the subtests “Social play” and “Dog alone” were coded by point sampling at 3-second intervals and then extrapolated to proportions of time (Table 4). Based on a subsample of dogs coded using both point sampling and absolute durations of behaviour, we determined sufficient agreement between the two measurement methods, justifying the use of point sampling. During subtests where a reward was inaccessible in the unsolvable task box, the absolute duration of interacting with the box (with low or high effort) was coded (Table 4). For detailed definitions of quantitatively coded variables, see Supplementary Table 2.

**Table 4:** Quantitatively coded variables (refer to supplementary table 2) for complete definitions.

Subtest	Variable	Scoring
**3. Social play** (Owner playing with the dog without toys while the toy is inaccessible on a shelf)	Dog focusing on the toy	Point sampling
	Dog focusing on the owner	Point sampling
**6. Dog alone** (The dog has access to the toy; owner and experimenter leave the room for 30 s)	Dog interacting with the toy	Point sampling
**10. Toy inaccessible (in a box)** (The dog’s toy is placed in the unsolvable task box, thus rendering it inaccessible. Food puzzle is freely accessible)	Dog interacting with the box with high effort (trying to enter the box - scratching with both paws, biting, pushing the box)	Duration
	Dog interacting with the box with low effort (no tendency to enter the box, sniffing, scratching with one paw)	Duration
	Dog interacting with food or food puzzle (even when the food has been consumed)	Duration
**13. Toy inaccessible (on a shelf)** (The dog’s toy is placed on a shelf out of the dog’s reach)	Dog focusing on the toy	Duration

## Questionnaire on addictive-like behaviours

A questionnaire was developed (available in English and German) (for inter- and intra-rater reliability based on over 1500 dogs recruited via an online survey; see Supplementary Table 6). The questions relating to behavioural addiction criteria relevant to the present manuscript are shown in Table 5. They were rated on a 5-point Likert scale indicating the extent of agreement with the statement (1 – strongly disagree; 2 – partly disagree; 3 – neither agree nor disagree; 4 – partly agree; 5 – strongly agree). The dog owners were asked to complete this questionnaire during the cool-down period of the behavioural test.

**Table 5:** Behavioural addiction criteria (cf^70^ and corresponding questions in our “Big Dog Reward And Motivation Questionnaire”.

Behavioural addiction criteria	Corresponding question
Salience	⋅ When a toy is available, my dog forgets everything around him/her ⋅ My dog loses interest in food when playing with a toy ⋅ My dog loses interest in social contact when playing with a toy ⋅ My dog will continue to play with a toy despite adverse consequences ⋅ My dog appears not to feel pain or discomfort while playing with a toy
Mood modification through carrying out the behaviour	⋅ Playing with a toy makes my dog happy
Craving	⋅ My dog cannot wait to play with a toy again when he/she has not had this opportunity for a while
Lack of self-control	When my dog plays with a toy, it is difficult to stop him/her My dog will continue to play with a toy despite adverse consequences
Tolerance	⋅ My dog requires more and more play to become satisfied
Withdrawal symptoms	⋅ When my dog is prevented from playing, he/she becomes stressed and agitated
Risk of relapse after abstinence*	⋅ (My dog cannot wait to play again when he/she has not had this opportunity for a while)

* under owner’s control.

## Analysis

SPSS Statistics Version 23 (IBM Corporation and its Licensors 1989, 2015) was used to compute a Categorical Principal Component Analysis and Mann-Whitney U tests. R version 4.1.0 (The R Foundation for Statistical Computing, 2021) was used to create boxplots and to calculate linear models.

### Assessment of differences in summary scores of individual behavioural addiction criteria between high-AB dogs and low-AB dogs

We calculated summary scores for the four addiction criteria for each subtest by summing up the points for each minute. Using Mann-Whitney U tests, we tested whether there was a difference in the addiction criteria Craving, Lack of self-control, Mood modification and Salience between dogs classified as high-AB dogs (AB-T score ≥ 44.2 points) and low-AB dogs (AB-T score < 44.2 points). Note that although components of Salience and craving were used to calculate the AB-T score, these are not identical to the 1 − 0 variables of Salience and craving here. While the addiction criteria were coded as 1/0 for each minute, the variables included in the AB-T score were more detailed, and individual elements potentially indicative of addictive-like behaviour were differentiated. Mood modification and Lack of self-control were not used in the designation of the AB-T score. See Sect. 1.6 and Table 3 for more details.

### Assessment of differences in durations of toy-directed behaviours between high-AB dogs and low-AB dogs

We performed Mann-Whitney U tests to assess whether high-AB and low-AB dogs differed in quantitatively coded variables such as time engaging with the toy during different subtests, attempting to attain an unavailable toy, etc. (see Supplementary Tables 4 and 5).

### Associations between questionnaire and behaviour test results and calculation of an Addictive-like Behaviour Questionnaire score (AB-Q score)

Linear models were used to assess associations between the addictive-like behaviour score and the 19 questionnaire questions targeting addictive-like behaviour. Model requirements were checked by visually assessing normality and homoscedasticity of the residuals. If applicable, the dependent variable was transformed.

Additionally, Mann-Whitney U tests were used to test whether the 19 questionnaire scores differed between high-AB and low-AB dogs. This was the case for fifteen questions; therefore, these were summed up to generate an Addictive-like Behaviour Questionnaire score (AB-Q score). Cohen’s R was used as a measure of effect size.

Both intra-rater reliability (available for 274 dogs, including dogs from the online survey) and inter-rater reliability (available for 24 dogs) of the AB-Q score were very good (see Supplementary Table 6).

Due to the exploratory nature of this study, no correction for multiple testing was performed (as recommended by^73^.

## Results

### Addictive-like behaviour test score (AB-T score)

The mid-point of the data range of the AB-T score was 44.2 (range 6.6–95). Therefore, dogs scoring 44.2 or higher were classified as showing a high tendency for addictive-like behaviour (high-AB dogs). This was the case for thirty-three of the 105 highly play-motivated dogs tested, with a mean score of 59.7 points and a median of 58.6. The mean AB-T score for low-AB dogs (< 44.2 points) was 23.1, and the median was 22.8. For descriptive statistics, see Supplementary Table 7.

### Assessment of differences between high-AB dogs and low-AB dogs in summary scores of individual behavioural addiction criteria

**Fig. 2:**
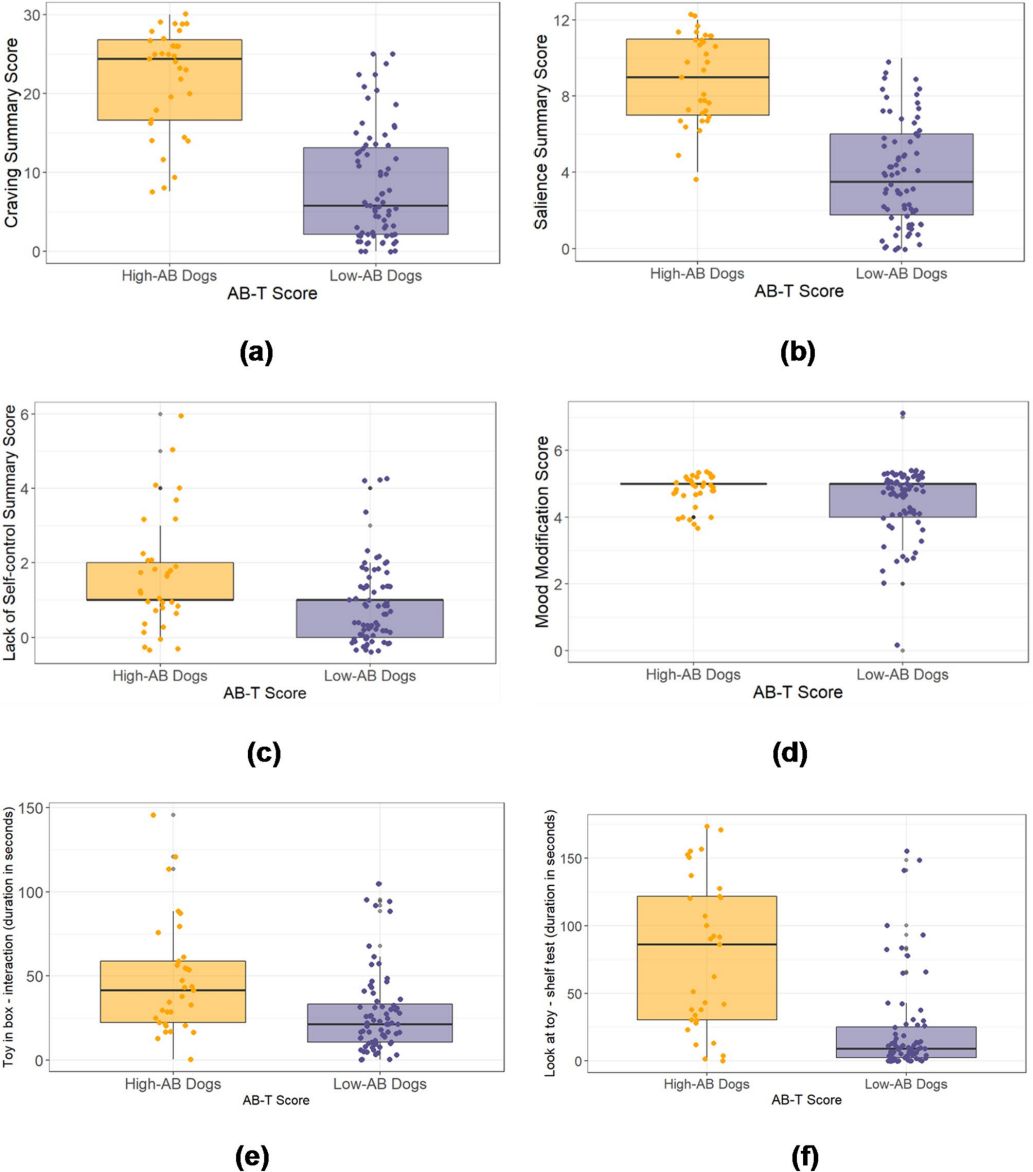
Behavioural addiction criteria and toy-directed behaviors: Multiple sub-figures (a–g) illustrate differences between high-AB and low-AB dogs across various metrics. **(a)** Craving summary score **(b)** Salience summary score **(c)** Lack of self-control summary score **(d)** Mood modification summary **(e)** Interaction with the box while the toy is enclosed – total interaction time (s) **(f)** Toy on the shelf– duration of looking at the toy (s).

Mann-Whitney U tests indicated that high-AB dogs scored significantly higher than low-AB dogs on craving (U = 217, *p* < 0.0001), salience (U = 208, *p* < 0.0001), and lack of self-control (U = 756.5, *p* = 0.002), but not mood modification (U = 1022, *p* = 0.157), in the behaviour test (see Supplementary Table 4, Figs. 2a-d). For descriptive statistics, see Supplementary Table 8.

### Quantitatively coded variables

High-AB dogs interacted significantly longer with the box than low-AB dogs in the ‘toy in the box’ subtest (U = 675.5, *p* < 0.0001). They also spent more time looking at the toy on the shelf during the ‘toy on shelf’ subtest (U = 414.5, *p* < 0.0001) and the ‘social play without toys’ subtest (U = 942.5, *p* = 0.021), while focusing less on the owner in the latter (U = 819.5, *p* = 0.011) compared to low-AB dogs (Supplementary Table 5, Fig. 2e and f). However, time spent interacting with the toy while the owner and experimenter were out of the room did not differ significantly between high-AB and low-AB dogs (U = 994, *p* = 0.135; Supplementary Table 5). For descriptive statistics, see Supplementary Table 9.

### Addictive-like behaviour questionnaire score (AB-Q score)

Linear models demonstrated significant associations between the AB-T score and 18 out of 19 individual questions (Table 6). However, according to Mann-Whitney U tests, only fifteen questions differed significantly between dogs classified as showing a high tendency for addictive-like behaviour in the behaviour test (AB-T score ≥ 44.2) and those that did not. These fifteen questions (Cohen’s *R* > 0.2 – see Table 6) were summed up into the Addictive-like Behaviour Questionnaire score (AB-Q score) (see Table 6).

**Table 6:** Association between the Addictive-like behaviour test score (AB-T score) and 19 behavioural addiction criteria questions and results of Mann-Whitney U tests comparing 19 behavioural addiction criteria questions between high-AB dogs and low-AB dogs.

	Linear Model results					Mann-Whitney U test results	
Question	Estimate	95% CI	*p*	Adjusted *R*^2^	Cohen’s *R*	U	*p*
My dog is obsessed with playing ball/fetching objects	6.23	3.66–8.79	< 0.0001	0.19	−0.28	643.5	0.006
If I did not counteract this tendency, my dog would be a ball junkie	5.38	3.12–7.65	< 0.0001	0.19	−0.38	525.5	< 0.001
My dog will continue-play with a ball/toy despite adverse consequences	5.75	3.19–8.30	< 0.0001	0.17	−0.34	559	0.001
My dog is obsessed with playing tug	5.68	2.99–8.37	< 0.0001	0.15	−0.42	467	< 0.001
My dog is tireless when it comes to playing ball/fetching objects	5.89	3.02–8.75	< 0.0001	0.14	−0.37	550	< 0.001
My dog is a ball junkie	4.65	2.29-7.00	0.0002	0.13	−0.24	682.5	0.017
When my dog plays with a ball/toy, it is difficult to stop him/her	5.53	2.65–8.41	0.0003	0.13	−0.25	675.5	0.015
With a ball/toy as a reward, I can recall my dog from any situation	5.67	2.64–8.71	0.0004	0.12	−0.25	679.5	0.016
My dog is so focused on playing ball/fetching objects that he/she hardly notices what is happening around him/her	5.33	2.47–8.19	0.0004	0.12	−0.26	663.5	0.011
My dog can be motivated by a ball/toy to do things that he/she is not keen on doing or tolerating otherwise (e.g. approaching a person/object that is frightening-him/her, getting in the car, etc.)	5.09	2.20–7.97	0.0007	0.11	−0.21	722	0.041
My dog loses interest in social contact when playing with a ball/toy	4.36	1.78–6.93	0.0012	0.11	−0.26	663.5	0.012
My dog loses interest in food while he/she is playing with a ball/toy	4.23	1.69–6.76	0.0013	0.11	−0.22	705.5	0.029
My dog gets more and more aroused while playing with a ball/toy	4.99	1.93–8.05	0.0017	0.09	−0.25	671	0.014
Constantly wants to initiate toy play	4.39	1.55–7.24	0.0028	0.08	−0.20	731	0.049
My dog cannot wait to play again when he/she has not had this opportunity for a while	4.03	1.32–6.73	0.0039	0.08	−0.21	726.5	0.044
My dog requires more and more play to become satisfied	4.53	0.82–8.24	0.0173	0.05	−0.23	707	0.026
My dog appears not to feel pain or discomfort while he/she is playing with the ball/toy	3.27	0.46–6.08	0.0232	0.04	−0.18	754.5	0.072
When my dog is prevented from accessing his/her toy, he/she becomes stressed and agitated	3.62	0.29–6.95	0.0336	0.04	−0.18	772	0.085
To what extent is your dog attached to his/her favourite object	1.85	−1.81-5.51	0.3171	0.0001	−0.09	870.5	0.396

## Discussion

This study represents the beginning of the exploration of addictive-like behaviour in domestic dogs. Convergent behavioural measures support the existence of an addictive-like behavioural phenotype in 33 of the 105 tested highly play-motivated dogs. Note that we specifically sought dogs exhibiting extreme behaviour; thus, this proportion is not a reflection of the general population. Perhaps not surprising, working breeds – many of which are known to have been artificially selected for high toy or predatory motivation^74–76^ – were overrepresented in the sample.

As predicted, dogs classified as high-AB dogs based on the detailed AB-T score (Addictive-like Behaviour Test score) scored significantly higher than low-AB dogs on the individual criteria craving, salience, and lack of self-control in the behaviour test. Contrary to the prediction, mood modification (when given access to a toy) did not differ between high and low-AB dogs. In retrospect, however, this lack of difference between the two groups strengthens our argument that we were measuring a phenotype beyond mere enjoyment of play. Still, despite the significant differences between high- and low-AB dogs in the other investigated addiction criteria, Salience, Craving and Loss of Self Control, there was generally high variation between individuals.

In line with the predictions, high-AB dogs showed higher durations of focusing on and trying to access an inaccessible toy than low-AB dogs, often prioritising attempting to access the toy over eating or interacting with the owner. Thus, there was general agreement between the three alternative methods of coding the data (detailed behaviour score, addiction criteria, and quantitative coding), indicating internal consistency.

The external validity of the behaviour test was demonstrated by significant associations of the AB-T score with 18 out of 19 questions from the addictive-like behaviour questionnaire filled in by the dogs’ owners, intended to measure addictive-like behaviour in everyday life. Nonetheless, although significant, the effect sizes were relatively low, indicating that no single question would have predictive value for assessing a tendency for addictive-like behaviour in dogs.

In studies using animal models of substance addiction, one way to differentiate an addiction from drug use that occurs due to lack of choice is to present the subject with a choice between the addictive substance and other highly desirable stimuli. If an individual continues to take the drug at the expense of these other options (such as consumption of a food reward), this points to the possibility of addictive-like behaviour^77,78^. Consistent with this, high-AB dogs showed a loss of interest in other relevant stimuli, focusing on the inaccessible toy and foregoing the opportunity to consume food or to engage with their owner. The latter is also reminiscent of behavioural addictions in humans, leading to a decline in social interactions^79^.

The intense toy-seeking and loss of interest in other stimuli, despite the availability of food or social interaction – considered as indicators for salience and persistence – might resemble “hyperfocus,” a trait associated with ADHD and autism in humans^80,81^. However, unlike typical hyperfocus, which often emerges in the absence of competing stimuli, dogs in our study were presented with alternative salient rewards (e.g., the toy was placed on a shelf while the owner actively invited the dog to engage in social play; in another subtest, food was available in a puzzle toy while the preferred toy was inaccessible in a closed container), and they still showed a preference for the inaccessible toy. Like dogs with ADHD, dogs in the current study with high AB-T scores in general exhibited high impulsivity (labelled as “loss of self-control”), and some individuals displayed heightened activity (which could be interpreted as the hyperactivity component of ADHD^64,65^ in particular during the cool-down period. Thus, further research is needed to explore commonalities and differences between addictive-like behaviour and ADHD-like behaviour in dogs. While dogs with a high tendency for addictive-like behaviour might exhibit many characteristics of dogs with ADHD, the converse is not necessarily true – dogs might show ADHD-like behaviour without displaying any hyperfixation on toys.

Another characteristic of addicted individuals is that they are willing to pursue their addiction even if it has adverse consequences^82^. In the current study, “adversity” was elicited by the owner and the experimenter leaving the room in order to assess the effect of social isolation on the behavioural addiction criteria. Isolation in an unfamiliar place is well-established as a stressful experience for dogs^83–86^. However, this subtest was not a good measure of addictive-like behaviour: Time spent interacting with the toy while the dog was alone did not differ significantly between high-AB and low-AB dogs. For welfare reasons, we decided against exposing the dogs to more severe stressors; however, it cannot be ruled out that this subtest was not “aversive” enough. The dog was left alone for only 30 seconds, and the subtest took place in the middle of the test when the dogs were already habituated to the test room. It is also possible that individual differences in subjects’ separation distress, independent of play motivation, affected the results. Additionally, there was no clear contingency between interacting with the toy and the ‘adverse’ outcome (owner leaving). Future studies could potentially enhance the design by providing the dog with an explicit choice, such as by placing the toy in a separate room, away from the owner and the experimenter. This could help determine whether the dog is willing to risk being alone in an unusual or new environment when it normally prefers the safety of being near its owner. Such a design would better reflect the conflict between competing motivations (social security vs. reward seeking) and could offer a more valid test of the criterion of persistence under adversity.

Still, the importance of continued efforts to engage in the behaviour despite adverse consequences was demonstrated in the questionnaire, where one of the highest associations with the AB-T score was found with the question, “My dog will continue to play with a ball/toy despite adverse consequences”. This suggests that some dogs may fulfil the criterion of continuing the addictive-like behaviour despite adverse consequences in real life, even if this could not be demonstrated in the behaviour test.

A critical factor in addiction is the propensity to attribute incentive salience to classically conditioned cues predicting rewards^87,88^. In humans, cues associated with addictive behaviours, such as specific locations or objects, can induce craving and drug administration^88,89^. In dogs, a toy such as a ball could represent such as a conditioned cue. It may achieve its value, for example, by the experience of chasing and catching. For many domestic dogs, balls or other toys possess incentive salience, according to the three criteria by Robinson and Berridge^49^: they (1) “elicit approach” (i.e. they become “wanted” and act as “motivational magnets”); (2) “they can energise ongoing actions by eliciting cue-triggered wanting”; (3) “they can act as reinforcers in their own right, reinforcing the acquisition of a new instrumental response (measurable by conditioned reinforcement)” (cf^49^, p. 3139].).

The perceived value of the toy was demonstrated in our study by many dogs having difficulty relinquishing the toy. It can be speculated that balls become ‘motivational magnets’ by being associated with species-typical predatory behaviour (cf^48^. The high salience of the toy was especially apparent in subtests where dogs were foregoing available alternatives such as freely available food or social play with the owner, at the expense of trying to regain their inaccessible toy.

In both rodents and humans^90,91^, individuals with a higher tendency to attribute incentive salience to classically conditioned cues predicting rewards (sign trackers) are more vulnerable to addiction than goal trackers, who focus primarily on the (location of the) reward itself^88,92^, see the meta-analysis by^93^. Tendency to sign-track vs. goal-track is associated with the risk of addiction and is also related to variations in the dopaminergic system and stress physiology^88^.

While it was not explicitly measured in the current study, in dogs, a tendency to sign track might be advantageous in a training context – i.e., maintaining motivation would be easier in dogs that are not only sensitive to rewards but also attribute value to the cues predicting these rewards, even if not always followed by a primary reinforcer. Sensitivity to reward – and propensity to attribute incentive salience to reward-predictive cues – would thus be highly relevant traits in relation to trainability and might be selected for especially in working dog breeds.

Several publications state the importance of ‘obsessive’ play motivation for working dog success^42,94–99^. Dogs with extreme toy motivation are believed to show better focus, reduced distractibility and greater trainability^97,99^. However, if such motivation becomes addictive-like, it needs to be questioned whether the well-being of these dogs may be compromised. If dogs prioritise toy interactions over other essential aspects of their daily lives this may have maladaptive effects, as is the case in humans with behavioural addictions^100,101^. Certainly, adverse health consequences may arise from repetitive ball chasing, like straining ligaments and joints^56^. Moreover, welfare would be compromised when dogs experience a high level of frustration when access to their reward is prevented (cf^102^.

Anecdotally, when play motivation becomes excessive, irritability, high arousal levels, and frustration may negatively affect dogs’ trainability and work^103^. Indeed, as stated by Mathews^96^, the high ‘drive’ of search dogs often makes them unsuitable as family pets, which is also supported by owner reports that pet dogs with extreme motivation for toys are often problematic to control^102^.

Thus, it needs to be questioned when play becomes maladaptive. Do high-AB dogs still ‘like’ to play, or have they progressed to primarily ‘wanting’ and the compulsive need to continue^104^?. Similar to the escalating engagement seen in human behavioural addictions^105^, some dogs would repeatedly spin, jump, focus or bark towards the unavailable toy on the shelf for the duration of the subtest. Two dogs even managed to destroy the box enclosing their favourite toy. These behaviours might be likened to the repetitive actions observed in individuals with behavioural addictions^41^. Nonetheless, such behaviours may also occur in other behavioural phenotypes such as canine compulsive disorder or autism spectrum-like behaviours^106^. Further research is needed to elucidate commonalities and differences between such phenotypes in dogs.

Behavioural addictions in humans often involve emotional dependency on specific activities^107^. Whether dogs similarly seek comfort, stimulation, or stress relief through persistent engagement with the toy could not be determined in the context of the behaviour test. In the questionnaire, “Is attached to the favourite toy” was the only question not significantly associated with the AB-T score. Thus, further research is required to determine whether dogs develop an emotional dependency on their toys (as described anecdotally).

To better understand the origin and possible functional underpinnings of excessive toy-directed behaviour in dogs, future research should examine whether similar patterns of excessive object play occur in non-domesticated canids. While data are limited, recent studies have shown that both hand-reared and wild wolf pups engage in object play^108^. For instance, wolf pups have been observed developing a preference for toys and spending increasing amounts of time with them over time^108^. Hand-reared wolf pups will even retrieve objects to humans^109^. In the wild, wolves have also been seen interacting with human-made objects^110^. These findings suggest that object play is not unique to dogs but rather could represent a broader trait shared by canids. Comparative studies are needed to assess how common and functionally relevant such behaviours are in wolves, which would help clarify the biological basis of the addictive-like behaviours observed in some dogs.

Being the first of its kind, this study has its limitations. As no gold standard exists, the study is exploratory, and our categorisation of dogs into high and low addictive-like behaviour groups, determined by a data range split, was somewhat arbitrary. Nonetheless, the assignment of high- and low-AB categories corresponded well to the first author’s personal assessment of addictive-like tendencies in the participant dogs.

In interpreting the questionnaire results, it is important to acknowledge the potential biases associated with using owner-reported questionnaires. Owners may unintentionally project their perceptions or expectations onto their dogs’ behaviours, potentially leading to discrepancies between reported and observed behaviours in behavioural test. This is particularly relevant in cases where owners have multiple dogs, as they are likely to compare their pets to one another, influencing their assessment, such as by underestimating or overestimating certain behaviours. For instance, an owner with a highly active dog may rate their less active dog as overly calm. Integrating owner reports and objective testing allows for a more comprehensive and accurate canine behaviour evaluation.

## )Conclusions

To conclude, there appear to be parallels between excessive toy motivation in dogs and behavioural addictions in humans. Interestingly, also in humans, the first officially recognised behavioural addictions (gambling and internet gaming) originate in play^28–30,111^. Generally, play is an activity that induces a pleasurable emotional state^6^. In humans, much evidence suggests that video games can affect people’s lives positively. They make players feel better about themselves, help raise their self-esteem and assist people in dealing with everyday stress^111^. Some people are excessive gamers, but only a minority would be classified as addicts^111–113^. Similarly, many dogs may greatly enjoy toy play without developing harmful compulsions (cf. in humans^28–30,111^).

Despite the observed parallels between high-AB dogs and humans affected by behavioural addictions, we refrain from conclusively characterising high-AB dogs as exhibiting addictive behaviour, given the absence of established benchmarks or standardised criteria. It is important to be cautious when pathologising behaviour, especially given that even in humans, addictive behaviours are still difficult to define and measure^114^. To further understand possible parallels in the processes underlying behavioural addictions in humans and excessive toy motivation in dogs, subsequent research endeavours should seek to correlate individual differences in addictive-like behaviour in dogs with characteristics associated with addictive behaviours in humans, such as high impulsivity, impaired reversal learning, heightened perseveration, and delayed extinction of previously rewarded responses^115,116^.

## Supplementary Information

Below is the link to the electronic supplementary material.

Supplementary Material 1

## Data Availability

All data supporting the findings of this study are available within the paper and its Supplementary Information.

## References

[1] Bradshaw, J. W. S., Pullen, A. J. & Rooney, N. J. Why do adult dogs ‘play’? *Behav. Process.* **110**, 82–87 (2015). 10.1016/j.beproc.2014.09.023 25251020

[2] Burghardt, G. M., Albright, J. D. & Davis, K. M. Motivation, development and object play: comparative perspectives with lessons from dogs. *Behaviour* **153**, 767–793 (2016).

[3] Diamond, J. & Behaviour, A. B. & undefined. A comparative analysis of social play in birds. *JSTORJ Diamond, AB BondBehaviour, 2003•JSTOR* (2003). (2003).

[4] Bateson, P. Play, playfulness, creativity and innovation. *Anim. Behav. Cognition*. **1**, 99–112 (2014).

[5] Sommerville, R., O’Connor, E. A. & Asher, L. Why do dogs play? Function and welfare implications of play in the domestic dog. *Appl. Anim. Behav. Sci.* **197**, 1–8 (2017).

[6] Held, S. D. E. & Špinka, M. Animal play and animal welfare. *Animal Behaviour* vol. 81 891–899 Preprint at (2011). 10.1016/j.anbehav.2011.01.007

[7] Bateson, P. Play Playfulness, creativity and innovation. *Anim Behav. Cogn* **1**(2), 99–112 (2014).

[8] Trezza, V., Damsteegt, R., Marijke Achterberg, E. J. & Vanderschuren, L. J. M. J. Nucleus accumbens µ-opioid receptors mediate social reward. *J. Neurosci.* **31**, 6362–6370 (2011). 21525276 10.1523/JNEUROSCI.5492-10.2011 PMC3098965

[9] Blois-Heulin, C. et al. Animal welfare: could adult play be a false friend?? *Anim. Behav. Cogn.* **2**, 156–185 (2015).

[10] Panksepp, J. *Affective Neuroscience: the Foundations of Human and Animal Emotions* (Oxford University Press, 1998).

[11] Dias, P. A. D. & Rangel-Negrín, A. Affiliative contacts and greetings. *The Int. Encyclopedia of Primatology,. Wiley-Blackwell, pp *1-4 (2016).

[12] Maroney, N., Williams, B. J., Thomas, A., Skues, J. & Moulding, R. A Stress-Coping model of problem online video game use. *Int. J. Ment Health Addict.* **17**, 845–858 (2019).

[13] Blasi, M. D. I. et al. Problematic video game use as an emotional coping strategy: evidence from a sample of MMORPG gamers. *J. Behav. Addict.* **8**, 25–34 (2019). 30739460 10.1556/2006.8.2019.02 PMC7044601

[14] Melodia, F., Canale, N. & Griffiths, M. D. The role of avoidance coping and escape motives in problematic online gaming: A systematic literature review. *Int. J. Ment Health Addict.* **20**, 996–1022 (2022).

[15] Organization., W. H. ICD-11. *ICD-11* (2019). https://icd.who.int/en

[16] Freimuth, M., Moniz, S. & Kim, S. R. Clarifying exercise addiction: differential diagnosis, co-occurring disorders, and phases of addiction. *Int. J. Environ. Res. Public. Health*. **8**, 4069–4081 (2011). 22073029 10.3390/ijerph8104069 PMC3210598

[17] Olsen, C. M. Natural rewards, neuroplasticity, and non-drug addictions. *Neuropharmacology* vol. 61 1109–1122 Preprint at (2011). 10.1016/j.neuropharm.2011.03.010 10.1016/j.neuropharm.2011.03.010 PMC3139704 21459101

[18] Alavi, S. S. et al. Behavioral addiction versus substance addiction: correspondence of psychiatric and psychological views. *Int. J. Prev. Med.* **3**, 290–294 (2012). 22624087 PMC3354400

[19] Pinna, F. et al. Behavioural addictions and the transition from DSM-IV-TR to DSM-5. *J. Psychopathol.* **21**, 380–389 (2015).

[20] Robbins, T. W. & Clark, L. Behavioral addictions. *Current Opinion in Neurobiology* vol. 30 66–72 Preprint at (2015). 10.1016/j.conb.2014.09.005 10.1016/j.conb.2014.09.005 25262209

[21] Fan, L., Li, K., Xin, J., Wang, Y. & Li, Y. Family Subjective Socioeconomic Status and University Students’ Online Shopping Addiction: A Gender-Based Analysis. https://home.liebertpub.com/cyber (2022). 10.1089/CYBER.2021.0344 10.1089/cyber.2021.0344 36472467

[22] Larocque, E. & Moreau, N. When sport is taken to extremes: A sociohistorical analysis of sport addiction. *Int. Rev. Sociol. Sport*. 10.1177/10126902221104956 (2022).

[23] Atroszko, P. A. Work addiction as a behavioural addiction: Towards a valid identification of problematic behaviour. *Australian and New Zealand Journal of Psychiatry* vol. 53 284–285 Preprint at (2019). 10.1177/0004867419828496 10.1177/0004867419828496 30754989

[24] Kun, B., Takacs, Z. K., Richman, M. J., Griffiths, M. D. & Demetrovics, Z. Work addiction and personality: A meta-analytic study. *J. Behav. Addict.* **9**, 945–966 (2020). 33361486 10.1556/2006.2020.00097 PMC8969726

[25] Niedermoser, D. W. et al. Shopping addiction: A brief review. *Pract. Innovations*. **6**, 199–207 (2021).

[26] American Psychiatric Association. Diagnostic and Statistical Manual of Mental Disorders. (2013). 10.1176/APPI.BOOKS.9780890425596

[27] Walia, B., Kim, J., Ijere, I. & Sanders, S. Video game addictive symptom level, use intensity, and hedonic experience: Cross-sectional questionnaire study. *JMIR Serious Games*. **10**, e33661 (2022). 35471995 10.2196/33661 PMC9227790

[28] Beranuy, M., Carbonell, X. & Griffiths, M. D. A qualitative analysis of online gaming addicts in treatment. *Int. J. Ment Health Addict.* **11**, 149–161 (2013).

[29] Higuchi, S. et al. Development and validation of a nine-item short screening test for ICD-11 gaming disorder (GAMES test) and Estimation of the prevalence in the general young population. *J. Behav. Addict.* **10**, 263–280 (2021). 34232907 10.1556/2006.2021.00041 PMC8996803

[30] Stevens, M. W. R., Dorstyn, D., Delfabbro, P. H. & King, D. L. Global prevalence of gaming disorder: A systematic review and meta-analysis. *Australian and New Zealand Journal of Psychiatry* vol. 55 553–568 Preprint at (2021). 10.1177/0004867420962851 10.1177/0004867420962851 33028074

[31] Di Segni, M., Patrono, E., Patella, L., Puglisi-Allegra, S. & Ventura, R. Animal models of compulsive eating behavior. *Nutrients* vol. 6 4591–4609 Preprint at (2014). 10.3390/nu6104591 10.3390/nu6104591 PMC4210935 25340369

[32] Brené S. et al. Running is rewarding and antidepressive.* Physiol. Behav.* **1-2**, 136–140 (2007). 10.1016/j.physbeh.2007.05.015 PMC2040025 17561174

[33] Kanarek, R. B., D’Anci, K. E., Jurdak, N. & Mathes, W. F. Running and addiction: precipitated withdrawal in a rat model of Activity-Based anorexia. *Behav. Neurosci.* **123**, 905–912 (2009). 19634951 10.1037/a0015896 PMC2786257

[34] Winstanley, C. A. Gambling rats: insight into impulsive and addictive behavior. *Neuropsychopharmacology* **36**, 359 (2011). 21116252 10.1038/npp.2010.136 PMC3055520

[35] Pitchers, K. K. et al. ∆FosB in the nucleus accumbens is critical for reinforcing effects of sexual reward. *Genes Brain Behav.* **9**, 831–840 (2010). 20618447 10.1111/j.1601-183X.2010.00621.x PMC2970635

[36] Kolb, E. M., Kelly, S. A. & Garland, T. Mice from lines selectively bred for high voluntary wheel running exhibit lower blood pressure during withdrawal from wheel access. *Physiol. Behav.* **112–113**, 49–55 (2013). 23458632 10.1016/j.physbeh.2013.02.010

[37] Carter, P. A., Swallow, J. G., Davis, S. J. & Garland, T. Nesting Behavior of House Mice (Mus Domesticus) Selected for Increased Wheel-Running Activity. *Behavior Genetics* 30, 85–94 (2000). (2000). 10.1023/a:1001967019229 10979598

[38] De Visser, L., Van Den Bos, R. & Spruijt, B. M. Automated home cage observations as a tool to measure the effects of wheel running on cage floor locomotion. *Behav. Brain. Res.* **160**, 382–388 (2005). 15863235 10.1016/j.bbr.2004.12.004

[39] Kas, M. J. H. & Edgar, D. M. A nonphotic stimulus inverts the diurnal-nocturnal phase preference in Octodon Degus. *J. Neurosci.* **19**, 328–333 (1999). 9870962 10.1523/JNEUROSCI.19-01-00328.1999 PMC6782376

[40] Reebs, S. G. & St-Onge, P. Running wheel choice by Syrian hamsters. *Lab. Anim.* **39**, 442–451 (2005). 16197712 10.1258/002367705774286493

[41] Grant, J. E., Potenza, M. N., Weinstein, A. & Gorelick, D. A. Introduction to behavioral addictions. *American Journal of Drug and Alcohol Abuse* vol. 36 233–241 Preprint at (2010). 10.3109/00952990.2010.491884 10.3109/00952990.2010.491884 PMC3164585 20560821

[42] Jamieson, L. T. J., Baxter, G. S. & Murray, P. J. Identifying suitable detection dogs. *Applied Animal Behaviour Science* vol. 195 1–7 Preprint at (2017). 10.1016/j.applanim.2017.06.010

[43] Rooney, N. J., Bradshaw, J. W. & Almey, H. Attributes of specialist search dogs—a questionnaire survey of UK dog handlers and trainers. *J. Forensic Sci.* **49**, 1–7 (2004). 15027550

[44] Naderi, S., Miklósi, Á., Dóka, A. & Csányi, V. Co-operative interactions between blind persons and their dogs. *Appl. Anim. Behav. Sci.* **74**, 59–80 (2001).

[45] Mariti, C. et al. Dog attachment to man: A comparison between pet and working dogs. *J. Veterinary Behav.* **8**, 135–145 (2013).

[46] Kolm, N., Temrin, H., Miklósi, Á. & Kubinyi, E. & Zsolt garamszegi, L. The link between selection for function and human-directed play behaviour in dogs. (2020). 10.1098/rsbl.2020.0366 10.1098/rsbl.2020.0366 PMC7532715 32961091

[47] Sundman, A. S., Johnsson, M., Wright, D. & Jensen, P. Similar recent selection criteria associated with different behavioural effects in two dog breeds. *Genes Brain Behav.* **15**, 750–756 (2016). 27520587 10.1111/gbb.12317

[48] Lazarowski, L. et al. Selecting Dogs for Explosives Detection: Behavioral Characteristics. *Frontiers in Veterinary Science* vol. 7 597 Preprint at (2020). 10.3389/fvets.2020.00597 10.3389/fvets.2020.00597 PMC7493654 33088829

[49] Robinson, T. E. & Berridge, K. C. The incentive sensitization theory of addiction: some current issues. *Philosophical Trans. Royal Soc. B: Biol. Sci.* **363**, 3137–3146 (2008). (The Royal SocietyLondon. 10.1098/rstb.2008.0093 PMC2607325 18640920

[50] Stephens, D. N. et al. Reward sensitivity: Issues of measurement, and achieving consilience between human and animal phenotypes. *Addiction Biology* vol. 15 146–168 Preprint at (2010). 10.1111/j.1369-1600.2009.00193.x 10.1111/j.1369-1600.2009.00193.x 20148777

[51] Antons, S., Brand, M. & Potenza, M. N. Neurobiology of cue-reactivity, craving, and inhibitory control in non-substance addictive behaviors. *Journal of the Neurological Sciences* vol. 415 116952 Preprint at (2020). 10.1016/j.jns.2020.116952 10.1016/j.jns.2020.116952 32534370

[52] Choi, J. S. et al. Dysfunctional inhibitory control and impulsivity in internet addiction. *Psychiatry Res.* **215**, 424–428 (2014). 24370334 10.1016/j.psychres.2013.12.001

[53] Kräplin, A. et al. The role of inhibitory control and decisionmaking in the course of internet gaming disorder. *J. Behav. Addict.* **9**, 990–1001 (2021). 10.1556/2006.2020.00076 PMC8969738 33136066

[54] Hebebrand, J. et al. ‘Eating addiction’, rather than ‘food addiction’, better captures addictive-like eating behavior. *Neuroscience and Biobehavioral Reviews* vol. 47 295–306 Preprint at (2014). 10.1016/j.neubiorev.2014.08.016 10.1016/j.neubiorev.2014.08.016 25205078

[55] Käufer, M. ‘Throw the damn ball!’ Warum Ballwerfen kein Spiel ist. in … *und weg ist er! Jagdverhalten und mögliche Alternativen* 129–154 (Filander, Erlangen, 2014).

[56] Marcellin-Little, D. J., Levine, D. & Taylor, R. Rehabilitation and conditioning of sporting dogs. *Veterinary Clinics of North America - Small Animal Practice* vol. 35 1427–1439 Preprint at (2005). 10.1016/j.cvsm.2005.08.002 10.1016/j.cvsm.2005.08.002 16260320

[57] Overall, K. L. Natural animal models of human psychiatric conditions: assessment of mechanism and validity. *Prog Neuropsychopharmacol. Biol. Psychiatry*. **24**, 727–776 (2000). 11191711 10.1016/s0278-5846(00)00104-4

[58] Boulougouris, V., Chamberlain, S. R. & Robbins, T. W. Cross-species models of OCD spectrum disorders. *Psychiatry Res.* **170**, 15–21 (2009). 19819024 10.1016/j.psychres.2008.07.016

[59] Vermeire, S. et al. Serotonin 2A receptor, serotonin transporter and dopamine transporter alterations in dogs with compulsive behaviour as a promising model for human obsessive-compulsive disorder. *Psychiatry Res. Neuroimaging*. **201**, 78–87 (2012). 10.1016/j.pscychresns.2011.06.006 22285716

[60] Hoffman, J. M., Creevy, K. E., Franks, A., O’Neill, D. G. & Promislow, D. E. L. The companion dog as a model for human aging and mortality. *Aging Cell.* **17**, e12737 (2018). 29457329 10.1111/acel.12737 PMC5946068

[61] Sándor, S. & Kubinyi, E. Genetic pathways of aging and their relevance in the dog as a natural model of human aging. *Front. Genet.* **10**, 948 (2019). 31681409 10.3389/fgene.2019.00948 PMC6813227

[62] Vitek, M. P. et al. Translational animal models for alzheimer’s disease: an alzheimer’s association business consortium think tank. *Alzheimer’s Dementia: Translational Res. Clin. Interventions*. **6**, e12114 (2020). 10.1002/trc2.12114 PMC7798310 33457489

[63] González-Martínez, Á., de Miguel, M., Graña, S., Costas, N., Diéguez, F. J. & X. & Serotonin and dopamine blood levels in ADHD-Like dogs. *Animals* **13**, 1037 (2023). 36978578 10.3390/ani13061037 PMC10044280

[64] Sulkama, S. et al. Canine hyperactivity, impulsivity, and inattention share similar demographic risk factors and behavioural comorbidities with human ADHD. *Transl Psychiatry*. **11**, 1–9 (2021). 34599148 10.1038/s41398-021-01626-x PMC8486809

[65] Csibra, B., Bunford, N. & Gácsi, M. Development of a human-analogue, 3-symptom domain dog ADHD and functionality rating scale (DAFRS). *Sci. Rep.* **14**, 1–18 (2024). 38245569 10.1038/s41598-024-51924-9 PMC10799898

[66] Salonen, M. et al. Personality traits associate with behavioral problems in pet dogs. *Transl Psychiatry* **12**, 1-7 (2022). 10.1038/s41398-022-01841-0 PMC8866408 35197456

[67] Tian, R. et al. Modeling SHANK3-associated autism spectrum disorder in Beagle dogs via CRISPR/Cas9 gene editing. *Molecular Psychiatry 2023 28:9* 28, 3739–3750 (2023). 10.1038/s41380-023-02276-9 37848710

[68] Li, Y. et al. Reduced attention to human eyes in autism-associated Shank3 mutant laboratory beagle dogs. *Mol. Psychiatry*. **30**, 3765–3773 (2025). 40148549 10.1038/s41380-025-02965-7

[69] Zhu, F., Shi, Q., Jiang, Y., hui, Zhang, Y. Q. & Zhao, H. Impaired synaptic function and hyperexcitability of the pyramidal neurons in the prefrontal cortex of autism-associated Shank3 mutant dogs. *Springer* 15, (2024). 10.1186/s13229-024-00587-4 PMC10829216 38297387

[70] Griffiths, M. Classification and treatment of behavioural addictions. *Nurs. Pract.* **82**, 44–46 (2015).

[71] Landolfi, E. Exercise addiction. *Sports Med.* **43**, 111–119 (2012). 10.1007/s40279-012-0013-x 23329605

[72] Van Rooij, A. J. & Prause, N. A critical review of internet addiction criteria with suggestions for the future. *J. Behav. Addict.* **3**, 203–213 (2014). 25592305 10.1556/JBA.3.2014.4.1 PMC4291825

[73] Bender, R. & Lange, S. Adjusting for multiple testing - When and how? *J. Clin. Epidemiol.* **54**, 343–349 (2001). 11297884 10.1016/s0895-4356(00)00314-0

[74] Coppinger, R. & Coppinger, L. *Dogs: A New Understanding of Canine Origin, Behavior, and Evolution* (University of Chicago Press, 2002).

[75] Mehrkam, L. R., Hall, N. J., Haitz, C. & Wynne, C. D. L. The influence of breed and environmental factors on social and solitary play in dogs (Canis lupus familiaris). *Learn. Behav.* **45**, 367–377 (2017). 28702755 10.3758/s13420-017-0283-0

[76] Eken Asp, H., Fikse, W. F., Nilsson, K. & Strandberg, E. Breed differences in everyday behaviour of dogs. *Appl. Anim. Behav. Sci.* **169**, 69–77 (2015).

[77] Ahmed, S. H., Lenoir, M. & Guillem, K. Neurobiology of addiction versus drug use driven by lack of choice. *Current Opinion in Neurobiology* vol. 23 581–587 Preprint at (2013). 10.1016/j.conb.2013.01.028 10.1016/j.conb.2013.01.028 23428657

[78] Golden, S. A. et al. Compulsive Addiction-like aggressive behavior in mice. *Biol. Psychiatry*. **82**, 239–248 (2017). 28434654 10.1016/j.biopsych.2017.03.004 PMC5532078

[79] Kuss, D. J., Louws, J. & Wiers, R. W. Online gaming addiction? Motives predict addictive play behavior in massively multiplayer online role-playing games. *Cyberpsychol Behav. Soc. Netw.* **15**, 480–485 (2012). 22974351 10.1089/cyber.2012.0034

[80] Ashinoff, B. K. & Abu-Akel, A. Hyperfocus: the forgotten frontier of attention. *Psychological Research 2019 85:1* 85, 1–19 (2019). 10.1007/s00426-019-01245-8 PMC7851038 31541305

[81] Grotewiel, M. M., Crenshaw, M. E., Dorsey, A. & Street, E. Experiences of hyperfocus and flow in college students with and without attention deficit hyperactivity disorder (ADHD). *Curr. Psychol.* **42**, 13265–13275 (2023).

[82] Vanderschuren, L. J., Minnaard, A. M., Smeets, J. A. & Lesscher, H. M. Punishment models of addictive behavior. *Curr. Opin. Behav. Sci.* **13**, 77–84 (2017).

[83] Palestrini, C., Previde, E. P., Spiezio, C. & Verga, M. Heart rate and behavioural responses of dogs in the ainsworth’s strange situation: A pilot study. *Appl. Anim. Behav. Sci.* **94**, 75–88 (2005).

[84] Palmer, R. & Custance, D. A counterbalanced version of ainsworth’s strange situation procedure reveals secure-base effects in dog-human relationships. *Appl. Anim. Behav. Sci.* **109**, 306–319 (2008).

[85] Ryan, M. G., Storey, A. E., Anderson, R. E. & Walsh, C. J. Physiological indicators of attachment in domestic dogs (Canis familiaris) and their owners in the strange situation test. *Front. Behav. Neurosci.* **13**, 456977 (2019). 10.3389/fnbeh.2019.00162 PMC6664005 31396061

[86] Riemer, S., Assis, L., Pike, T. W. & Mills, D. S. Dynamic changes in ear temperature in relation to separation distress in dogs. *Physiol. Behav.* **167**, 86–91 (2016). 27609307 10.1016/j.physbeh.2016.09.002

[87] Flagel, S. B. et al. An Animal Model of Genetic Vulnerability to Behavioral Disinhibition and Responsiveness to Reward-Related Cues: Implications for Addiction. *Neuropsychopharmacology 2010 35:2* 35, 388–400 (2009). 10.1038/npp.2009.142 PMC2794950 19794408

[88] Flagel, S. B., Akil, H. & Robinson, T. E. Individual differences in the attribution of incentive salience to reward-related cues: Implications for addiction. *Neuropharmacology* vol. 56 139–148 Preprint at (2009). 10.1016/j.neuropharm.2008.06.027 10.1016/j.neuropharm.2008.06.027 PMC2635343 18619474

[89] Marlatt, G. A. Cue exposure and relapse prevention in the treatment of addictive behaviors. *Addict. Behav.* **15**, 395–399 (1990). 2248112 10.1016/0306-4603(90)90048-3

[90] Olney, J. J., Warlow, S. M., Naffziger, E. E. & Berridge, K. C. Current perspectives on incentive salience and applications to clinical disorders. *Curr. Opin. Behav. Sci.* **22**, 59–69 (2018). 29503841 10.1016/j.cobeha.2018.01.007 PMC5831552

[91] Cofresí, R. U., Bartholow, B. D. & Piasecki, T. M. Evidence for incentive salience sensitization as a pathway to alcohol use disorder. *Neuroscience and Biobehavioral Reviews* vol. 107 897–926 Preprint at (2019). 10.1016/j.neubiorev.2019.10.009 10.1016/j.neubiorev.2019.10.009 PMC6878895 31672617

[92] Berridge, K. C., Robinson, T. E. & Aldridge, J. W. Dissecting components of reward: ‘liking’, ‘wanting’, and learning. *Current Opinion in Pharmacology* vol. 9 65–73 Preprint at (2009). 10.1016/j.coph.2008.12.014 10.1016/j.coph.2008.12.014 PMC2756052 19162544

[93] Meyer, P. J. et al. Quantifying individual variation in the propensity to attribute incentive salience to reward cues. *PLoS One*. **7**, e38987 (2012). 22761718 10.1371/journal.pone.0038987 PMC3382216

[94] Beebe, S. C., Howell, T. J. & Bennett, P. C. Using scent detection dogs in conservation settings: A review of scientific literature regarding their selection. *Front. Veterinary Sci.* **3** (1), Preprintathttpsdoiorg103389fvets201600096 (2016). 10.3389/fvets.2016.00096 PMC5083854 27840815

[95] Reed, S. E., Bidlack, A. L., Hurt, A. & Getz, W. M. Detection distance and environmental factors in conservation detection dog surveys. *J. Wildl. Manage.* **75**, 243–251 (2011).

[96] Mathews, F. et al. Effectiveness of search dogs compared with human observers in locating Bat carcasses at wind-turbine sites: A blinded randomized trial. *Wildl. Soc. Bull.* **37**, 34–40 (2013).

[97] Hsu, Y. & Serpell, J. A. Development and validation of a questionnaire for measuring behavior and temperament traits in pet dogs. *J. Am. Vet. Med. Assoc.* **223**, 1293–1300 (2003). 14621216 10.2460/javma.2003.223.1293

[98] Hsu, Y. & Sun, L. Factors associated with aggressive responses in pet dogs. *Appl. Anim. Behav. Sci.* **123**, 108–123 (2010).

[99] Serpell, J. A. & Hsu, Y. Effects of breed, sex, and neuter status on trainability in dogs. in *Anthrozoos* vol. 18 196–207 (2005).

[100] Müller, A. et al. Food addiction and other addictive behaviours in bariatric surgery candidates. *Eur. Eat. Disorders Rev.* **26**, 585–596 (2018). 10.1002/erv.2629 30094889

[101] Tunney, R. J. & James, R. J. E. Criteria for conceptualizing behavioural addiction should be informed by the underlying behavioural mechanism. *Addiction* **112**, 1720–1721 (2017). 28497595 10.1111/add.13831

[102] Gerencsér, L., Bunford, N., Moesta, A. & Miklósi, Á. Development and validation of the canine reward responsiveness scale -Examining individual differences in reward responsiveness of the domestic dog. *Sci Rep* **8**, 1-14 (2018). 10.1038/s41598-018-22605-1 PMC5849691 29535396

[103] Lindsay, S. R. *Handbook of applied dog behavior and training*. *Vol. 3: Procedures and protocols. Ames (IA): Iowa Stat University Press* (2005).

[104] Berridge, K. C. & Robinson, T. E. Liking, wanting, and the incentive-sensitization theory of addiction. *Am. Psychol.* **71**, 670–679 (2016). 27977239 10.1037/amp0000059 PMC5171207

[105] Grant, J. E., Brewer, J. A. & Potenza, M. N. The neurobiology of substance and behavioral addictions. *CNS Spectr.* **11**, 924–930 (2006). 17146406 10.1017/s109285290001511x

[106] Jacques, C. et al. What interests young autistic children? An exploratory study of object exploration and repetitive behavior. *PLoS One*. **13**, e0209251 (2018). 30596684 10.1371/journal.pone.0209251 PMC6312372

[107] Brand, M., Young, K. S., Laier, C., Wölfling, K. & Potenza, M. N. Integrating psychological and neurobiological considerations regarding the development and maintenance of specific Internet-use disorders: An Interaction of Person-Affect-Cognition-Execution (I-PACE) model. *Neuroscience and Biobehavioral Reviews* vol. 71 252–266 Preprint at (2016). 10.1016/j.neubiorev.2016.08.033 10.1016/j.neubiorev.2016.08.033 27590829

[108] Davis, K. M., Partin, A. M., Springer, C. M. & Burghardt, G. M. The development of object play in Wolf puppies (Canis lupus). *Int. J. Play.* **12**, 20–39 (2023).

[109] Hansen Wheat, C. & Temrin, H. Intrinsic Ball Retrieving in Wolf Puppies Suggests Standing Ancestral Variation for Human-Directed Play Behavior. *iScience* 23, 100811 (2020). 10.1016/j.isci.2019.100811 PMC7033638 31956066

[110] Ausband, D. E. Wolf use of humanmade objects during pup-rearing. *Anim. Behav. Cogn.*10.26451/abc.08.03.06.2021 (2021).

[111] Griffiths, M. Online computer gaming: advice for parents and teachers. *Educ. Health*. **27**, 3–6 (2009).

[112] Griffiths, M. D. *Diagnosis and Management of Video Game Addiction MMORPGs View Project Diagnosis and Management of Video Game Addiction*. (2008).

[113] Ko, C. H., Yen, J. Y., Yen, C. F., Chen, C. S. & Wang, S. Y. The association between internet addiction and belief of frustration intolerance: the gender difference. *CyberPsychology Behav.* **11**, 273–278 (2008). 10.1089/cpb.2007.0095 18537496

[114] Petry, N. M., Zajac, K. & Ginley, M. K. Behavioral addictions as mental disorders: to be or not to be? *Annu. Rev. Clin. Psychol.* **14**, 399–423 (2018). 29734827 10.1146/annurev-clinpsy-032816-045120 PMC5992581

[115] Everitt, B. J. et al. Neural mechanisms underlying the vulnerability to develop compulsive drug-seeking habits and addiction. *Philosophical Trans. Royal Soc. B: Biol. Sci.* **363**, 3125 (2008). 10.1098/rstb.2008.0089 PMC2607322 18640910

[116] Volkow, N. D. & Fowler, J. S. Addiction, a disease of compulsion and drive: involvement of the orbitofrontal cortex. *Cereb. Cortex*. **10**, 318–325 (2000). 10731226 10.1093/cercor/10.3.318

