# Supplementary material for: Addictive-like behavioural traits in pet dogs with extreme motivation for toy play
Source: Sci Rep. 2025 Oct 9;15:32613. doi: 10.1038/s41598-025-18636-0 (PMC12511593; doi:10.1038/s41598-025-18636-0)
Supplement: Supplementary file 1 — Supplementary Material 1 [file 41598_2025_18636_MOESM1_ESM.docx]

**Addictive-like behavioural traits in pet dogs with extreme motivation for toy play**

Alja Mazzini^1^, Katja Senn^1^, Federico Monteleone^2^, Stefanie Riemer^1,3*^

^1^ Division of Animal Welfare, Veterinary Public Health Institute, Vetsuisse Faculty, University of Bern, 3012 Bern, Switzerland

^2^ Division of Behavioural Ecology, Institute of Ecology and Evolution, University of Bern, 3032 Hinterkappelen, Switzerland

^3^ Messerli Research Institute, Department of Interdisciplinary Life Sciences, Vetmeduni Vienna, 1210 Vienna, Austria

^*^Corresponding author [Stefanie.Riemer@vetmeduni.ac.at](mailto:Stefanie.Riemer@vetmeduni.ac.at)

**Supplementary information**

**Supplementary Table 1:** Demographic information about the dogs participating in the study

| **ID** | **Breed** | **Age (years)** | **Sex** | **Neuter status** |
| --- | --- | --- | --- | --- |
| BC1 | Border Collie | 3.3 | F | Intact |
| BC2 | Border Collie | 3.5 | F | Intact |
| BC3 | Border Collie | 8.8 | F | Neutered |
| BC4 | Border Collie | 8.6 | M | N/A |
| BC5 | Border Collie | 4.5 | M | Intact |
| BC6 | Border Collie | 2.9 | M | Neutered |
| BC7 | Border Collie | 2.4 | M | Intact |
| BC8 | Border Collie | 4.7 | M | Neutered |
| BC9 | Border Collie | 4.2 | M | Intact |
| AS1 | Australian Shepherd | 6 | M | Neutered |
| BC/AS1 | Border Collie/Australian Shepherd Mix | 7.8 | M | Neutered |
| SHE1 | Sheltie | 5.5 | M | Neutered |
| SC1 | Smooth Collie | 3.2 | M | Neutered |
| AWK1 | Australian Working Kelpie | 9.6 | M | Neutered |
| BT1 | Tervueren | 1.2 | F | Intact |
| BT2 | Tervueren | 9.6 | F | Intact |
| BT3 | Tervueren | 5.7 | F | Intact |
| BT4 | Tervueren | 2.1 | M | Intact |
| BT5 | Tervueren | 4.3 | M | Neutered |
| BM/BT1 | Malinois/ Tervueren | 5.3 | M | Neutered |
| BM1 | Malinois | 2.2 | F | Intact |
| BM2 | Malinois | 2.4 | F | Intact |
| BM3 | Malinois | 5.2 | F | Neutered |
| BM4 | Malinois | 6.4 | F | Neutered |
| BM5 | Malinois | 8.3 | F | Neutered |
| BM6 | Malinois | 8.2 | F | Neutered |
| BM7 | Malinois | 6.6 | F | Neutered |
| BM8 | Malinois | 1.5 | F | Neutered |
| BM9 | Malinois | 2.6 | F | Neutered |
| BM10 | Malinois | 2 | M | Chemical castration (chip) |
| BM11 | Malinois | 5.2 | M | Intact |
| BM12 | Malinois | 8.5 | M | Intact |
| BM13 | Malinois | 8.5 | M | Intact |
| BM14 | Malinois | 4.4 | M | Intact |
| BM15 | Malinois | 8 | M | Neutered |
| BM16 | Malinois | 6.4 | M | Neutered |
| BM17 | Malinois | 3.3 | M | Neutered |
| BM18 | Malinois | 9.7 | M | Neutered |
| BMX1 | Malinois Mix | 10.1 | F | Neutered |
| BM/GSD1 | Malinois/German Shepherd Mix | 1.2 | M | Chemical castration (chip) |
| GSD1 | German Shepherd | 2.9 | F | Intact |
| GSD2 | German Shepherd | 7.2 | F | Neutered |
| GSD3 | German Shepherd | 5.2 | M | Intact |
| GSD4 | German Shepherd | 5.7 | M | Neutered |
| OGS1 | Old German Shepherd | 3.4 | M | Intact |
| DS1 | Dutch Shepherd | 3 | M | Intact |
| BEC1 | Beauceron | 7 | M | Intact |
| WSSD1 | White Swiss Shepherd Dog | 8.6 | M | Neutered |
| JRT1 | Jack Russell Terrier | 7 | M | Neutered |
| JRT2 | Jack Russell Terrier | 1.8 | F | Intact |
| JRT3 | Jack Russell Terrier | 8.3 | F | Neutered |
| JRT4 | Jack Russell Terrier | 1.9 | M | Neutered |
| JRT5 | Jack Russell Terrier | 8.9 | M | Neutered |
| PRT1 | Parson Russell Terrier | 6.7 | M | Neutered |
| PRT2 | Parson Russell Terrier | 3.4 | F | Intact |
| PRT3 | Parson Russell Terrier | 10.3 | M | Neutered |
| ST1 | Scottish Terrier | 6.2 | M | Neutered |
| ST2 | Scottish Terrier | 2.3 | F | Neutered |
| ST3 | Scottish Terrier | 1.3 | M | Intact |
| BOT1 | Border Terrier | 7.6 | M | Intact |
| BOT2 | Border Terrier | 5.4 | M | Intact |
| BOS1 | Boston Terrier | 3.9 | M | Intact |
| SFT1 | Smooth fox terrier | 3.1 | F | Neutered |
| GHT1 | German Hunting Terrier | 6 | M | Neutered |
| BUT1 | Bull Terrier | 6.3 | F | Neutered |
| BUT/X1 | Bull Terrier Mix | 2.8 | F | Neutered |
| LR1 | Labrador | 1.9 | F | Intact |
| LR2 | Labrador | 5 | F | Neutered |
| LR3 | Labrador | 2.1 | M | Chemical castration (chip) |
| LR4 | Labrador | 2.5 | F | Neutered |
| LR5 | Labrador | 5.2 | F | Neutered |
| LR6 | Labrador | 7.1 | F | Neutered |
| LR7 | Labrador | 1.9 | F | Intact |
| LR8 | Labrador | 3 | F | Neutered |
| LR9 | Labrador | 3.1 | M | Chemical castration (chip) |
| NSDTR1 | Nova Scotia Duck Tolling Retriever | 7.5 | M | Intact |
| NSDTR2 | Nova Scotia Duck Tolling Retriever | 1.4 | M | Intact |
| NSDTR3 | Nova Scotia Duck Tolling Retriever | 8.3 | F | Neutered |
| NSDTR4 | Nova Scotia Duck Tolling Retriever | 2.9 | M | Intact |
| FCR1 | Flat-Coated Retriever | 5.3 | M | Neutered |
| GR1 | Golden Retriever | 4.2 | F | Neutered |
| LR/GR1 | Labrador/Golden Retriever Mix | 1.8 | F | Intact |
| PB/X1 | Pitbull Mix | 7.3 | F | Neutered |
| RTB/X1 | Russian Tsvetnaya Bolonka Mix | 3.4 | F | Neutered |
| LR/BC1 | Labrador/Border Collie Mix | 5.2 | F | Neutered |
| RATT1 | Rottweiler | 5.2 | M | Neutered |
| LAND1 | Landseer | 8.7 | F | Neutered |
| DOB1 | Dobermann | 2.3 | M | Intact |
| MIX1 | Mix | 4.2 | F | Neutered |
| MIX2 | Mix | 9.7 | F | Neutered |
| MIX3 | Mix | 2.8 | M | Chemical castration (chip) |
| MIX4 | Mix | 5.1 | F | Neutered |
| TMIX1 | Terrier Mix | 7.6 | F | Neutered |
| HH1 | Hanover Hound | 4 | M | Neutered |
| GS1 | Giant Schnauzer | 7.9 | F | Neutered |
| LR/SP1 | Labrador/Standard Poodle Mix | 4 | M | Neutered |
| SP1 | Standard Poodle | 4.8 | F | Intact |
| LAR1 | Lagotto Romagnolo | 1.3 | M | Neutered |
| TT1 | Tibetan Terrier | 4.7 | M | Intact |
| LR/GR/SP1 | Labrador/Golden Retriever/Standard Poodle Mix | 4.5 | F | Neutered |
| RTB1 | Russian Tsvetnaya Bolonka | 8.8 | M | Neutered |
| PWD1 | Portuguese Water Dog Mix | 3.4 | F | Neutered |
| BC/APP1 | Border Collie/Appenzeller Mix | 9.2 | F | Neutered |
| BC/APP2 | Border Collie/Appenzeller Mix | 4.3 | M | Neutered |
| LR/AS/BS1 | Labrador/Australian Shepherd/Bergamasco Shepherd Mix | 7.6 | F | Neutered |

Among the 21 excluded dogs, multiple working breeds were represented (e.g. Australian Shepherd, Border Collie, Malinois, Labrador Retriever, German Shepherd), as well as non-working and mixed breeds (e.g. Rhodesian Ridgeback, Miniature Bull Terrier, Jack Russell Terrier, and various mixes). While the reasons for exclusion were diverse (see Methods), there was no clear pattern indicating systematic dropout associated with specific breed groups.

**Supplementary Table 2:** Detailed definitions of quantitatively coded variables

| Interact with box – low effort | Interact actively with the box with low effort (nose or paw is within 5 cm of the box). Scratch box with one paw or slight nudge of the box. Also, code if the dog circles the box for ≤ 10 seconds with the nose/paw further away than 5 cm. |
| --- | --- |
| Interact with box – high effort | Interact actively with the box with high effort. Bite at, paw at or scratch box with two paws or push box. Intermittent bouts of "low effort" interactions in between "high effort" interactions are coded as "high effort" if they don't last longer than one second. |
| Interact with food | Dog interacts actively with food puzzle; the dog's nose or paw is within 5 cm of food puzzle. Also, interacting with pieces of kibble or chewing. It is coded any time the dog interacts with the food (puzzle), even if the food has been consumed. |
| Focus on toy during social play without toys and when toy is inaccessible on a shelf | Dog's gaze is directed at the toy on the shelf. |
| Focus on owner during social play without toys | Dog's gaze is directed at the owner and is engaging with the owner |
| Interact with the toy when left alone | The sum of "dog inactive with the toy" (the dog is sitting/lying/standing with toy in his mouth) and "move with toy" (the dog is running/walking around the room with toy in his mouth). |

**Supplementary Table 3:** ICC absolute agreement, single measures two-way mixed-effects model, as a measure of inter-rater reliability for durations and scores measured in the behaviour test.

| **Subtest** | **Variable** | **Absolute agreement** |
| --- | --- | --- |
| Toy on shelf | Duration of gazing at the toy on the shelf | 0.995 |
| Toy in box | Duration of interaction with box | 0.999 |
| Social play | Focus on the toy during social play with the owner | 1.0 |
| Social play | Focus on the owner during social play with the owner | 0.850 |
| Dog alone | Dog interacting with the toy | 0.915 |
| All applicable subtests | Craving score (sum) | 0.975 |
| All applicable subtests | Salience score (sum) | 0.919 |
| All applicable subtests | Mood modification score (sum) | 0.869 |
| All applicable subtests | Lack of self-control score (sum) | 0.825 |

**Supplementary Table 4:** Results of Mann Whitney U tests comparing four behavioural addiction criteria between high-AB dogs and low-AB dogs

| **Dependent variable** | **U** | **p** |
| --- | --- | --- |
| Craving summary score | 217 | <0.0001 |
| Salience summary score | 208 | <0.0001 |
| Lack of self-control summary score | 756.5 | 0.002 |
| Mood modification summary score | 1022 | 0.157 |

**Supplementary Table 5:** Results of Mann Whitney U tests comparing quantitatively coded variables between high-AB dogs and low-AB dogs

| **Dependent variable** | **U** | **p** |
| --- | --- | --- |
| Toy in the box – interaction | 675.5 | <0.0001 |
| Look at the toy on the shelf | 414.5 | <0.0001 |
| Social play without toys – focus on the owner | 819.5 | 0.011 |
| Social play without toys – focus on the toy | 942.5 | 0.021 |
| Experimenter and owner out – dog interacting with the toy | 994 | 0.135 |

*Intra-rater and inter-rater reliability for the "Big Play and Motivation Questionnaire for Dogs"*

Besides including the dogs participating in the behaviour test, the questionnaire was distributed online via social media. The owners of 1.697 dogs (English: n=542 and German: n=1155) filled in the questionnaire (publication in prep.). This sample was used to assess inter- and intra-rater reliability. At least two months after the questionnaire was returned, emails were sent out to owners who had voluntarily provided their email addresses, inviting them to complete the questionnaire a second time for the same dog and/ or to share the questionnaire with a spouse/ relative/ friend who knows the dog well. Inter- and intra-rater reliability for the AB-Q score were analysed using ICC, absolute agreement, single measures, two-way mixed effects, as computed in IBM SPSS Statistics Version 23 (IBM Corporation and its licensors 1989, 2015) (**Supplementary Table 6**).

**Supplementary Table 6:** ICC absolute agreement, single measures two-way mixed-effects model as a measure of inter-rater and intra-rater reliability for the AB-Q score.

The questionnaire was returned by owners of 1.697 dogs (English: n=542 and German: n=1155) from 33 countries. The mean age of dogs was 5.28 years (SD = 3.41), and the sample included 383 intact males, 338 intact females, 436 neutered males, and 517 neutered females. Dogs that were chemically castrated (e.g., castration chip) were classified as NA. We did not enquire about the owners' demographics.

|  | **N** | **ICC** |
| --- | --- | --- |
| **Intra-rater reliability** | 274 | 0.892 |
| **Inter-rater reliability** | 24 | 0.834 |

**Supplementary Table 7:** Descriptive statistics for the AB-T score.

|  | Full sample | High-AB dogs (N=33) | Low-AB dogs (N=105) |
| --- | --- | --- | --- |
| Min. | 6.6 | 44.3 | 6.6 |
| Max. | 95 | 95 | 43.7 |
| Median | 32.2 | 58.6 | 22.8 |
| Inter-quartile range | 30.6 | 12.5 | 20.325 |
| Mean | 34.6 | 59.7 | 23.1 |
| Standard deviation | 20.6 | 12.6 | 11.2 |

**Supplementary Table 8:** Descriptive statistics comparing summary scores of individual behavioural addiction criteria in high-AB dogs and low-AB dogs

| Craving summary score | | | |
| --- | --- | --- | --- |
|  | **Full sample** | **High-AB dogs** | **Low-AB dogs** |
| Max. number of points | 30 | | |
| Mean | 12.51 | 21.77 | 8.25 |
| Median | 11.60 | 24.40 | 5.80 |
| Standard deviation | 9.35 | 6.64 | 7.06 |
| Salience summary score | | | |
| Max. number of points | 14 | | |
| Mean | 5.45 | 8.97 | 3.83 |
| Median | 5.00 | 9.00 | 3.5 |
| Standard deviation | 3.52 | 2.24 | 2.74 |
| Lack of self-control summary score | | | |
| Max. number of points | 6 | | |
| Mean | 1.12 | 1.73 | 0.85 |
| Median | 1.00 | 1.00 | 1.00 |
| Standard deviation | 1.27 | 1.53 | 1.03 |
| Mood modification summary score | | | |
| Max. number of points | 5 | | |
| Mean | 4.58 | 4.79 | 4.49 |
| Median | 5.00 | 5.00 | 5.00 |
| Standard deviation | 0.86 | 0.42 | 0.99 |

**Supplementary Table 9:** Descriptive statistics comparing quantitatively coded variables in high-AB dogs and low-AB dogs

| Toy in box – interaction (3 min) | | | |
| --- | --- | --- | --- |
|  | **Full sample** | **High-AB dogs** | **Low-AB dogs** |
| Mean | 34.19 | 48.28 | 27.73 |
| Median | 26.00 | 41.60 | 21.64 |
| Standard deviation | 28.81 | 33.54 | 23.97 |
| Look at the toy on the shelf (3 min) | | | |
| Mean | 40.36 | 79.02 | 22.69 |
| Median | 13.80 | 86.40 | 9.00 |
| Standard deviation | 50.02 | 55.60 | 35.49 |
| Social play without toys – focus on the owner | | | |
| Max. number of points | 20 | | |
| Mean | 11.49 | 9.73 | 12.29 |
| Median | 12.00 | 10.33 | 13.00 |
| Standard deviation | 4.86 | 4.43 | 4.86 |
| Social play without toys – focus on the toy | | | |
| Max. number of points | 20 | | |
| Mean | 0.91 | 1.70 | 0.56 |
| Median | 0.00 | 0.50 | 0.19 |
| Standard deviation | 2.45 | 3.41 | 1.77 |
| Experimenter and owner out – dog interacting with the toy | | | |
| Max. number of points | 10 | | |
| Mean | 1.92 | 2.30 | 1.75 |
| Median | 0.00 | 0.95 | 0.53 |
| Standard deviation | 2.92 | 3.03 | 2.87 |
